# An Electronic Pre-Exposure Prophylaxis Initiation and Maintenance Home Care System for Nonurban Young Men Who Have Sex With Men: Protocol for a Randomized Controlled Trial

**DOI:** 10.2196/13982

**Published:** 2019-06-10

**Authors:** Aaron J Siegler, James B Brock, Christopher B Hurt, Lauren Ahlschlager, Karen Dominguez, Colleen F Kelley, Samuel M Jenness, Gretchen Wilde, Samuel B Jameson, Gina Bailey-Herring, Leandro A Mena

**Affiliations:** 1 Department of Behavioral Sciences and Health Education Rollins School of Public Health Emory University Atlanta, GA United States; 2 Division of Infectious Diseases Department of Medicine University of Mississippi Medical Center Jackson, MS United States; 3 Institute for Global Health & Infectious Diseases University of North Carolina at Chapel Hill Chapel Hill, NC United States; 4 Department of Epidemiology Rollins School of Public Health Emory University Atlanta, GA United States; 5 Division of Infectious Diseases Department of Medicine Emory University School of Medicine Atlanta, GA United States; 6 Department of Population Health Science John D Bower School of Population Health University of Mississippi Medical Center Jackson, MS United States

**Keywords:** pre-exposure prophylaxis, sexual and gender minorities, prevention, smartphone, mobile apps, telemedicine, telehealth, mHealth

## Abstract

**Background:**

Pre-exposure prophylaxis (PrEP) is highly efficacious for preventing HIV but has not yet been brought to scale among at-risk persons. In several clinical trials in urban areas, technology-based interventions have shown a positive impact on PrEP adherence. In rural and small-town areas in the United States, which often do not have geographically proximal access to PrEP providers, additional support may be needed. This may be particularly true for younger persons who are more likely to face multiple barriers to accessing PrEP services. Home-based care, accomplished through a tailored mobile phone app, specimen self-collection (SSC), and interactive video consultations, could increase both PrEP initiation and persistence in care.

**Objective:**

The goal of this study is to assess the initiation and persistence in PrEP care for those randomized to a home-care intervention (electronic PrEP, ePrEP) relative to those assigned to the standard of care (control) condition. We will conduct additional assessments, including quantitative and qualitative analyses, to contextualize trial results and facilitate scale-up.

**Methods:**

This 2-arm, randomized controlled trial will enroll young men who have sex with men (YMSM) aged between 18 and 24 years from rural areas of Georgia, Mississippi, and North Carolina. The trial will seek to recruit a diverse sample, targeting 50% participation among highly impacted groups of black or Latino men who have sex with men. Intervention participants will receive a study app that incorporates a messaging platform, a scheduling and milestone-based tracking system for PrEP care progress, electronic behavioral surveys, and interactive video consultations with a clinician. Complemented by SSC kits mailed to laboratories for standard PrEP-related monitoring, the ePrEP system will allow participants to access PrEP care without leaving their homes. YMSM randomized to the control condition will receive a listing of nearest local PrEP providers to receive standard PrEP care. Both groups will complete quarterly electronic surveys. The primary outcome, assessed at 6 and 12 months after randomization, will be the difference in the proportion of intervention versus control participants that achieve protective levels of the active metabolite of oral PrEP (tenofovir diphosphate in dried blood spots).

**Results:**

Enrollment will begin in May 2019, with study completion in 2022.

**Conclusions:**

This trial will determine whether home PrEP care provided through an app-based platform is an efficacious means of expanding access to PrEP care for a diverse group of YMSM in rural and small-town areas of the United States.

**Trial Registration:**

ClinicalTrials.gov NCT03729570; https://clinicaltrials.gov/ct2/show/NCT03729570 (Archived by WebCite at http://www.webcitation.org/78RE2Qizf)

**International Registered Report Identifier (IRRID):**

PRR1-10.2196/13982

## Introduction

Men who have sex with men (MSM) continue to account for the majority of new HIV diagnoses in the United States [1]. HIV prevalence among MSM in rural counties is high, with many counties exceeding 15% prevalence [2]. Oral emtricitabine/tenofovir disoproxil fumarate (FTC/TDF) was approved by the US Food and Drug Administration (FDA) for HIV pre-exposure prophylaxis (PrEP) in 2012. Large-scale clinical and pragmatic trials of oral FTC/TDF demonstrated high efficacy and effectiveness in preventing sexual HIV acquisition when taken consistently [3-5], yet a national database of PrEP-prescribing clinics developed by the authors indicates a substantial lack of providers in rural areas [6]. There are a number of rural areas in the United States in which residents live in *PrEP deserts*, with no nearby PrEP-prescribing clinics [7]. An estimated 38,000 MSM eligible for PrEP would require a ≥2-hour round trip drive to their nearest clinic; over 100,000 would require ≥1-hour round trip drive to access PrEP services [7].

Within the elevated risk group of MSM, black MSM and young MSM (YMSM) aged between 15 and 24 years bear disproportionate burdens of incident HIV infection [1] and represent priority populations for interventions. Several successful technology-based interventions have been shown in clinical trials to increase PrEP adherence [8,9], yet these efforts have been conducted predominately in urban areas. The premise for this study is that a tailored approach for rural YMSM, addressing known barriers of transportation, access to knowledgeable providers, and privacy, is likely to yield demonstrable improvements in initiation of PrEP and persistence in PrEP care.

The focus of this project is the delivery of home-based PrEP care. Such a strategy has the potential to quickly make PrEP services available to MSM in rural areas who currently lack access. Using a mobile phone app, participants assigned to the intervention may initiate PrEP and be retained in follow-up care without leaving their home. This will be achieved through the *electronic PrEP (ePrEP)* system that combines app-based surveys and screening tools, interactive video consultations, and home specimen self-collection (SSC).

The primary aim of this efficacy trial is to determine the impact of the ePrEP system on the levels of PrEP protection achieved by MSM interested in initiating PrEP in rural and small-town areas. The hypothesis is that a higher proportion of ePrEP intervention participants will achieve biomarker-determined protective levels of tenofovir diphosphate (TFV-DP) relative to those randomized to standard of care (control). Secondary aims include conducting additional assessments to contextualize trial results, including adjusted analyses of the primary outcome, as well as cost effectiveness and cost-utility analyses.

## Methods

### Study Design

PrEP-naïve MSM will be recruited into the study, with black and Latino MSM to comprise half of all participants. Initial screening will be conducted online; eligible individuals will complete an electronic consent (Multimedia Appendix 1) and a baseline survey (Multimedia Appendix 2), and then will receive an SSC kit to verify clinical appropriateness of oral FTC/TDF use. The home SSC kit will comprise supplies needed to produce microtube and dried blood spot (DBS) samples for central laboratory testing, detailed instructions, and directions for mailing specimens to the laboratory. MSM determined to be eligible, including clinical eligibility to receive oral FTC/TDF as PrEP, will be enrolled and randomized to either intervention or control conditions. Randomization, conducted using the study’s electronic data capture (EDC) system, will be stratified by study site to decrease the likelihood of type I error because of the expected association between covariates and the primary study outcome.

**Figure 1:**
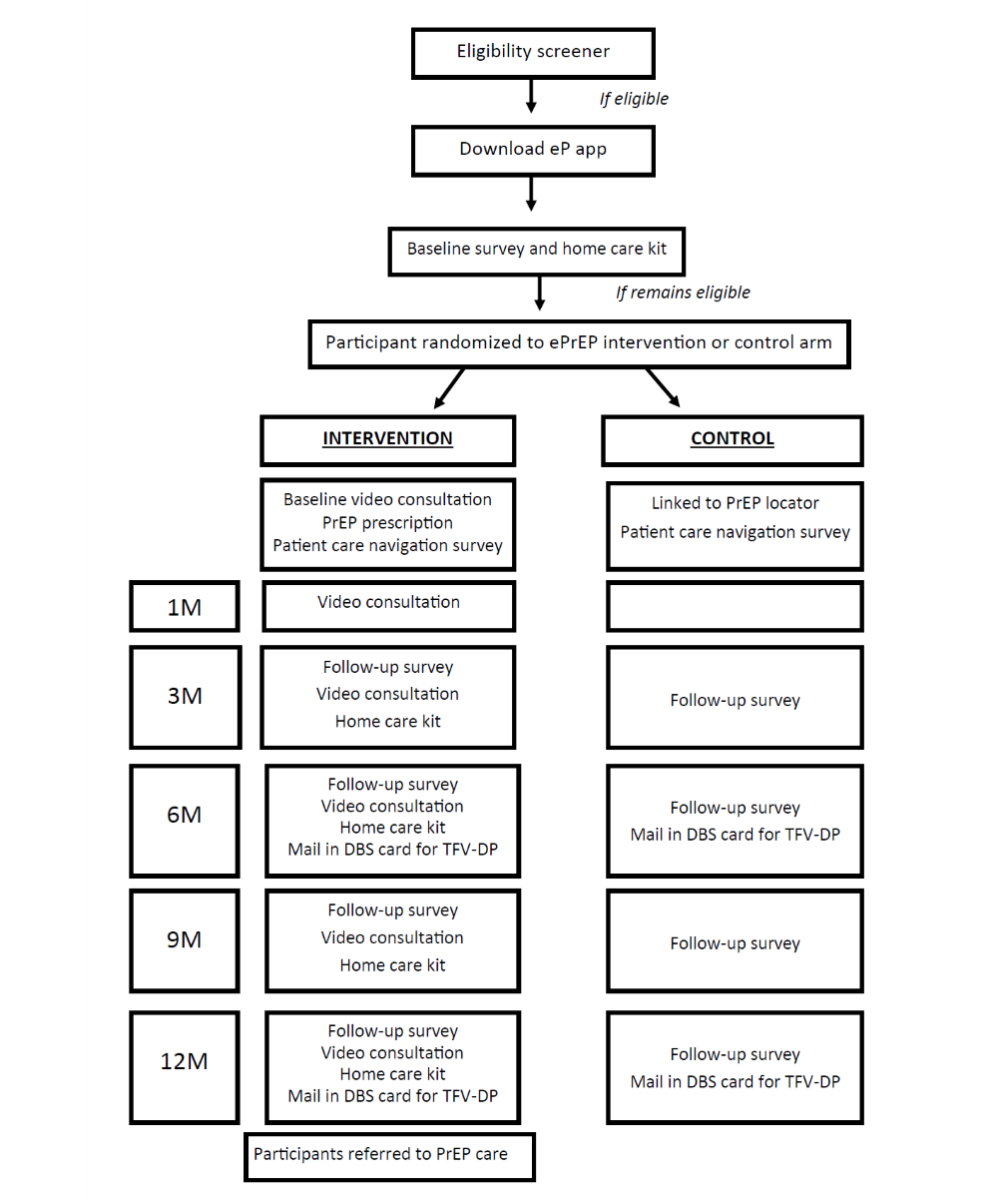
Electronic pre-exposure prophylaxis (ePrEP) study flow chart. DBS: dried blood spot; M: month; TFV-DP: tenofovir diphosphate.

Those assigned to the intervention ePrEP condition (n=120) will have an app-based interactive video consultation with a site study clinician who will prescribe PrEP as indicated [10]. At months 1, 3, 6, 9, and 12 postrandomization, participants complete *virtual* study visits that include app-based surveys, video consultations, and quarterly SSC for laboratory testing (per current practice guidelines) [11]. Participants assigned to the control condition (n=120) will be referred to a publicly available website that geolocates the nearest PrEP provider, using PrEP Locator [6]. All participants will mail-in self-collected DBSs for the determination of the primary outcome assessment of TFV-DP at month 12 postrandomization. All participants will also complete electronic surveys for secondary outcome assessment at baseline and months 3, 6, 9, and 12 postrandomization (Figure 1).

### Study Population and Recruitment

We will randomize 240 participants in Georgia, North Carolina, and Mississippi. Target recruitment is 50% among highly impacted groups of black or Latino MSM. To be eligible, a potential participant must (1) have been assigned male sex at birth, (2) be aged between 18 and 24 years, (3) live in a rural or small-town zip code based on the Centers for Disease Control and Prevention’s (CDC) urbanicity classifications [12], (4) provide informed consent, (5) be able to complete study consent and survey processes in English, (6) be willing to provide complete contact information (including 2 alternate contacts), (7) be able and willing to provide identification for verification, (8) own an iOS or Android mobile phone capable of running the study app, (9) be willing to use study-provided PrEP financial navigation services, (10) be willing to self-collect specimens, (11) be HIV-uninfected, as determined by laboratory testing using an antigen/antibody combination assay, (12) have a serum creatinine level that suggests creatinine clearance ≥60 mL/min, as determined by the Cockcroft-Gault equation, (13) be hepatitis B virus uninfected, as determined by laboratory testing, (14) have an indication for PrEP based on current guidance, including consideration of epidemic conditions [11], and (15) be interested in and willing to take daily FTC/TDF. Potential participants are excluded from enrollment if they (1) are currently taking oral FTC/TDF as PrEP, (2) have a medical contraindication to the use of FTC/TDF, (3) have symptoms of acute HIV infection, (4) are currently enrolled in any HIV prevention trials, (5) have a self-reported acquired or inherited disorder preventing the normal clotting of blood, such as hemophilia, or (6) have insurance coverage through a closed network system in which study clinicians cannot prescribe. Individuals with signs of acute HIV infection who are HIV antigen/antibody negative may, upon the discretion of study clinicians based on additional laboratory testing and/or discussion with the participant, be determined to be eligible for the study. Due to the additional clinical follow-up needed for persons with hepatitis B infection, we excluded those testing positive from this telemedicine study.

Primary participant recruitment will be conducted using banner advertisements and brief electronic messages on geospatial networking apps and social media platforms. Our target of 50% recruitment of highly impacted groups will be sought through study advertisement placement and content strategies. Although the target is not a hard cap, we anticipate that we will be able to meet it based on past recruitment experience and will seek alternative recruitment strategies as needed. Recruitment, led by the Emory Center for AIDS Research, which has led similar work previously [13-15], will target ads to nonurban zip codes that would indicate potential eligibility for the study. Secondary methods for recruiting participants, such as peer referral, will be used if needed.

### Screening, Consent, and Enrollment Procedures

Individuals responding to electronic ads will be directed to a secure electronic platform to view a brief introductory statement, consent to be screened, and complete a brief eligibility screener. Screener-eligible participants will provide personal contact information and complete the full study consent by using mouse drag/touchscreen features to capture and document their signature. Study staff will review name, contact information, and other data, such as internet protocol address, for ineligible individuals to detect any instances of rescreening. Staff will call participants to answer any questions they might have about the study and to reassess screener eligibility. Eligible and consenting participants will download the study app on their personal mobile phone from an *app store* and create a personal, password-protected app account. Both intervention and control participants will use research-assessment components of the app (eg, access to the survey platform and reminders); however, only intervention participants will have access to the app components relevant to PrEP care. The baseline survey will be immediately available for completion in the app, and the staff will receive an automated notification to mail an SSC kit to the participant to determine study eligibility. Participants will be enrolled in the trial and assigned a randomization code, if they return the SSC kit and complete the required study surveys within 1 month and the laboratory test results indicate eligibility. If these activities are not completed within 1 month, participants will be asked to repeat the appropriate previous step. Throughout the consent and enrollment process, study staff will be available by email and by phone during study hours to answer participants’ questions. Study recruitment and retention activities will be conducted centrally by the staff at Emory University.

### Intervention

The ePrEP intervention, developed based on Andersen behavioral model adapted to HIV care (ABMH), is designed to ease the burden of initiating and maintaining PrEP care by providing home SSC kits and telemedicine through a custom-built app for patients and Web portal for clinicians.

#### Theoretical Model

**Figure 2:**
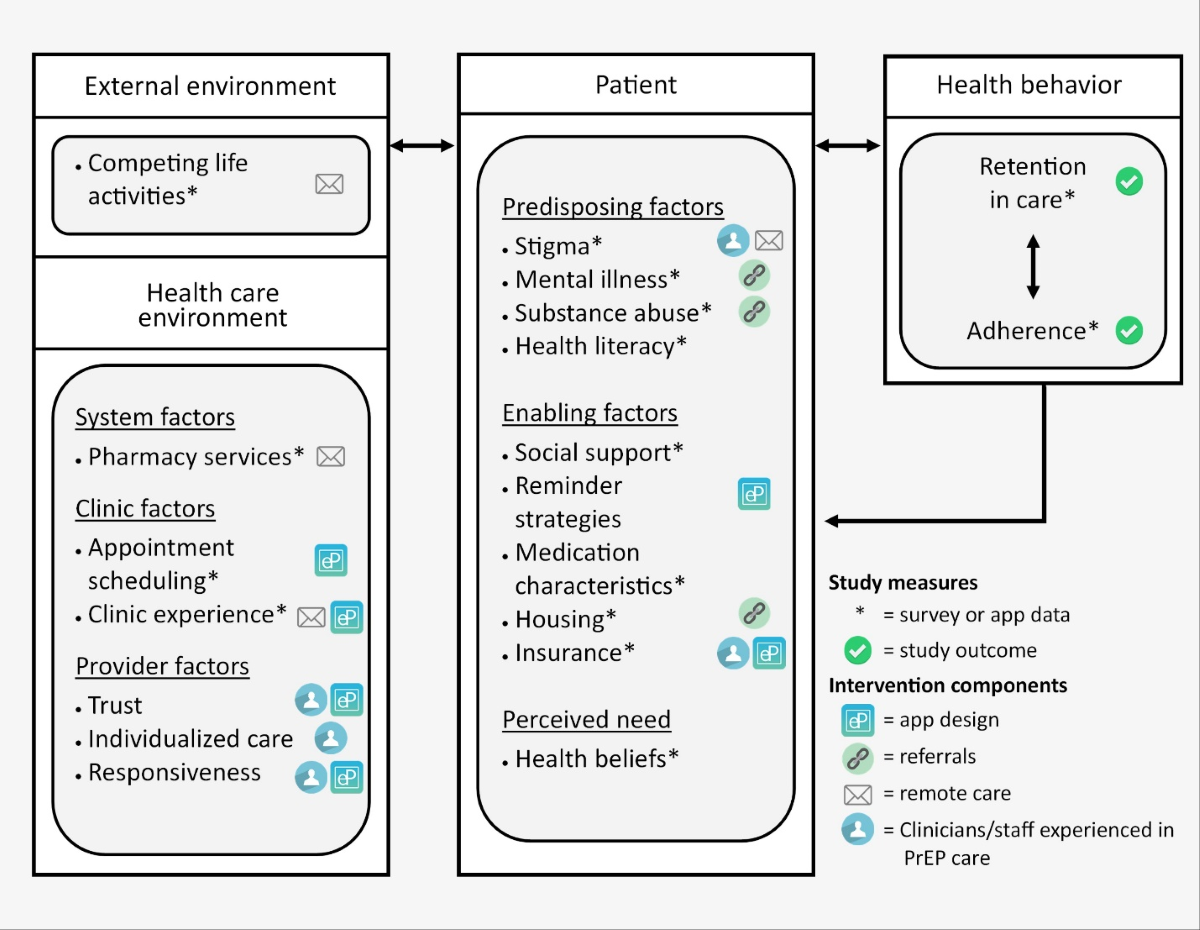
Electronic pre-exposure prophylaxis (ePrEP) intervention and study measures in Andersen behavioral model.

ABMH explicitly incorporates the biomedical nature of the intervention and also directly addresses patient experiences in health care and lived environments [16,17]. This multilevel model, with a focus on health care settings, describes how environmental and patient-level factors impact both retention in care and medication adherence (Figure 2). ABMH informs the development of a number of intervention components, such as an app-based scheduler to lower barriers to making appointments, an app-based message portal to increase provider responsiveness, use of PrEP-experienced pharmacies to facilitate PrEP prescriptions, use of interactive video consultations with lesbian, gay, bisexual, transgender, queer-friendly clinicians to facilitate provider trust, and referral strategies to address predisposing factors such as substance abuse or mental health problems. The model informs intervention assessment plans, including selection of appropriate measures and construction of multivariate models to assess intervention performance.

#### Specimen Self-Collection Kit

To allow for laboratory testing for PrEP care and for the study outcome, ePrEP will use an SSC kit that has previously been pilot tested [18]. Participants will receive a plain box via standard mail that includes video and written instructions, materials for each specimen to be collected, and a call-in help line. After specimen collection, participants will enclose them in a prepaid mailer to be sent directly to a study laboratory. On the basis of the results from the pilot assessment, we anticipate that most participants will be able to self-collect specimens. Our study laboratory will perform a visual assessment for the sufficiency of self-collected specimens based on the study requirements for quantity (eg, enough blood in a card spot) and quality (eg, properly sealed containers). For specimens determined to be insufficient or with indeterminate results, we will give participants an option of completing a second SSC or receiving a referral to a local health department or a commercial laboratory. Testing of all specimens will be performed at Clinical Laboratory Improvement Amendments–certified laboratories, with costs covered by the study. FDA-approved tests will be used for all tests used for PrEP care.

#### Study App

**Figure 3:**
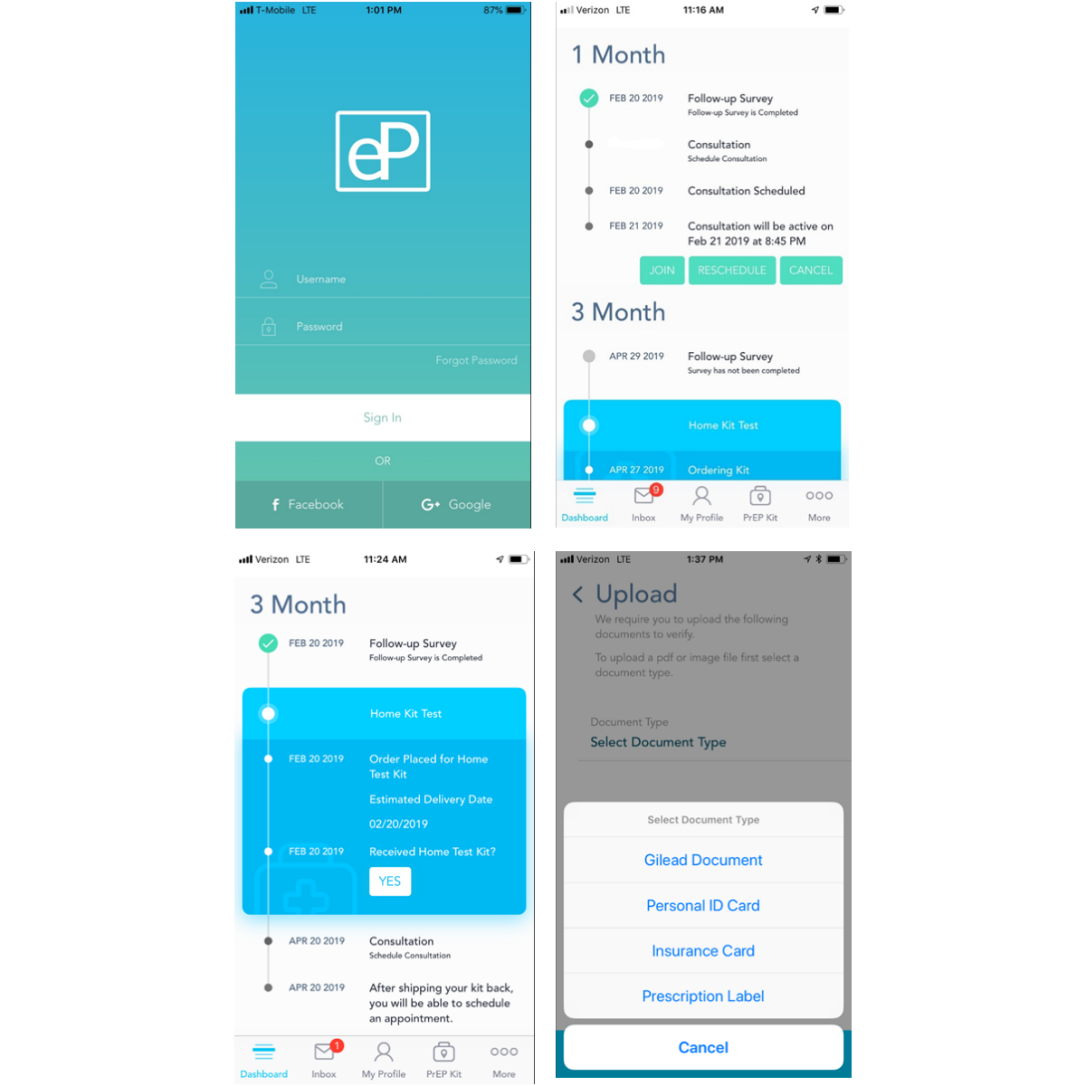
Screenshots of the eP app.

The study app, generically named *eP* to protect participant privacy on their mobile phones, will be the primary participant-facing component of the intervention. eP will be structured around a *dashboard* timeline (Figure 3) that visually depicts successful completion of past steps (green *check* marks), proximal future steps that require immediate action (activity buttons), and distal future steps needed for maintenance in ePrEP care (grey dots next to an anticipated future due date). The adaptive dashboard will refresh as tasks are completed. For instance, once an SSC is returned, scheduling of the next interactive video consultation will become available. An *inbox* screen (bottom navigation bar) will feature a variety of automated messages for required actions, reminders, and updates to facilitate the participant’s progress in ePrEP care. This messaging system also allows for custom messages to be sent to and from study staff and clinicians. A *profile* screen will allow participants to alter their personal information, such as changing their preferred pharmacy or home shipping address, and a *PrEP kit* screen will allow participants to track shipping status of their SSC.

To initiate PrEP care, electronic surveys embedded in the eP app will collect behavioral information recommended by current practice guidelines [11]. Photo upload functionality will be used for the communication of documents such as those needed for financial navigation of access to PrEP, such as insurance- or employment-related documents required for assistance or copayment programs. Interactive video consultations with study clinicians will be scheduled by patients through an electronic calendaring function. Initiated in the dashboard, the consultations will be conducted on a secure, encrypted and Health Insurance Portability and Accountability Act (HIPAA)–compliant video platform within eP. A secure *inbox* text message system will increase access to and streamline communication with the clinical care team regarding questions such as medication side effects.

When participants initially download eP, they will only be able to access app information relevant to the overall research process. Once randomized, the intervention group will be able to see and use all eP components. A modified version of the app will be used by control participants, which includes only components applicable to research participation such as processes around the primary and secondary outcomes data collection. Both eP app versions will be hosted in a secure environment that uses Single Socket Layer technology for encryption of online information transfer. Access to all systems will require authentication through individual log-ins and passwords, and all data will be stored on secure and HIPAA-compliant servers.

#### Administration/Clinical Portals

Study staff and clinicians will manage participant interactions through website *portals* developed for the study that use a cloud platform to exchange data with the participant-facing eP app. Both administration and clinician platforms will share a number of key features, including automatic alerts when activity is required, such as a patient needing an additional reminder to schedule their video consultation. A secure text message platform will allow staff and clinicians to send messages to the participant’s eP inbox. The administrative portal will allow for tracking of the participant’s progress in completing research-related activities, including visualizations of each participant’s movement through milestones essential to successful study retention. The clinical portal will allow clinicians to view clinically relevant information for study participants, including lab results, self-reported sexual behaviors, and self-reported PrEP adherence and side effects. Clinicians will specify their available time slots for the interactive video consultations in an electronic calendaring function. The clinical portal will allow the collection of nuanced care information during consultations—general assessments, adherence assessments, referrals, prescriptions (PrEP and other as needed), and notes. Clinicians will also be able to use the portal to track pre-existing medical conditions and concomitant medications, as well as provide referrals for treatment as needed, including sexually transmitted infections (STI), HIV, and others such as mental health or substance use disorders. Finally, the clinical portal will be used by the clinicians to track and monitor the progress of each participant in PrEP care, including the results of SSC kits and scheduling of future consultations. The portal is not currently connected to electronic medical records for each study site, although such functionality could be added later if the trial indicates utility of the intervention.

### Statistical Analysis

#### Primary Outcome

The primary outcome measure will be protective levels of the active metabolite of oral PrEP (TFV-DP) drug levels at the 12-month study visit, using an intention-to-treat analysis. Using a pharmacological model developed in a cohort study of FTC/TDF use among MSM and transgender women [19], the concentration of TFV-DP as determined from a DBS can be used to infer the mean number of days per week FTC/TDF is ingested over approximately 1 month preceding specimen collection. The threshold used for the primary outcome measure will be a TFV-DP concentration considered to be a surrogate for substantial HIV protection: >700 fmol/punch, a level indicating ≥4 doses per week [20]. The intervention efficacy measure will be quantified as the difference in proportions of the intervention arm with this outcome compared with the control arm.

#### Secondary Outcomes

We will assess initiation into PrEP care based on self-report and pill bottle photos. Maintenance in PrEP care at the study midpoint will be assessed with the 6-month TFV-DP drug level measure. We will also track changes in PrEP indication, determined by the CDC and United States Public Health Service guidelines, over the course of each participant’s involvement in the study. Other secondary outcome measures will be harmonized with Adolescent Medicine Trials Network for HIV/AIDS Interventions (ATN) measures, when possible. Demographic, socioeconomic, and sexual behavioral risk measures are derived from the National HIV Behavioral Surveillance (NHBS) instrument and our previous research instruments [10,21]. Adherence will be assessed with self-reported number of pills taken in the past week. Safety outcomes will include acute HIV symptoms assessed with a checklist of 17 symptoms and criteria identified by Braun [22] and the presence of more common adverse effects identified on the medication label. A number of domains of the study’s theoretical model will be assessed to explore the context of the intervention performance: PrEP perceptions and PrEP use adapted from NHBS and other sources [10,23], depression [24], illicit and nonprescription drug use [25], sexual stigma [26], HIV severity and risk perceptions [27], HIV knowledge [25], medication adherence self-efficacy [28], the Systems Usability Scale [29,30], insurance coverage [14], use of social and geosocial networking sites [14], and the depth of clinician-patient relationship [31,32]. We will also seek to understand rationales for those who fail to persist in PrEP, assessing perceptions of PrEP barriers and concerns [33,34].

#### Power Estimates

Power analyses assumed 80% power to detect a difference at a 2-sided 5% significance level, using a 2-sample comparison of proportions. On the basis of retention in our past trials, we assumed a 20% attrition rate in both arms, with calculations assuming independent censoring. If 5% of participants in the control arm are above cut point levels of the outcome measure (TFV-DP), we will have sufficient power to detect a minimum detectable effect size of 13% absolute difference in the outcome measure (eg, ≥18% of the intervention participants have TFV-DP above cut point threshold). To allow for the detection of a scenario where intervention outcome proportion is equal to control+20% absolute increase, our study would remain sufficiently powered with any control participant uptake level ≤15%.

#### Statistical Analysis Plan

Logistic regression and log-linear models will be used to estimate the association between the intervention arm and primary study outcome, TFV-DP level. If prognostic factors associated with the outcome remain insufficiently balanced through stratified randomization, we may adjust for these factors in our models. Potential confounding factors that may be included are medication self-efficacy and motivation to take PrEP. Per protocol assessments that account for potential changes in intervention as delivered will be conducted. For instance, this would account for participants assigned to the intervention condition opting to instead receive standard of care PrEP from a nonstudy clinician.

A number of secondary analyses will be conducted using regression models. Intervention impact on the secondary outcomes of PrEP initiation and PrEP persistence will be determined using analogous regression models. We will model log_10_ TFV-DP levels as the outcome variable to potentially detect significant smaller, subclinical, differences in adherence between the intervention and control study arms. An additional analysis will account for the changes in each participant’s PrEP eligibility over time, per CDC guidelines. The primary outcome measure and its intent-to-treat analysis assume daily oral PrEP dosing with FTC/TDF. Medications indicated for PrEP, as well as commonly prescribed PrEP dosing, may change over the course of the study. Informed by new approvals and developments, we will measure self-reported medication use and dosing strategy, and a secondary analysis will be performed to account for the influence of these factors on study outcomes. Specific and aggregate measures of safety will be assessed, including renal function, HIV incidence, and incident bacterial STI.

Exploratory analyses of intervention effectiveness across subgroups and analysis of potential mediators of initiation or persistence in PrEP care across both study arms, such as self-efficacy will also be conducted. Understanding variables associated with success or failure in the intervention will inform future research and potentially guide clinician recommendations or policy regarding bringing remote PrEP care to scale. For participants who seroconvert during the study, levels of TFV-DP, PrEP persistence, and other clinically relevant study data will be analyzed.

#### Cost-Effectiveness and Cost-Utility Analyses

We will employ standard methods of cost analyses as recommended by the U S Panel on Cost-Effectiveness in Health and Medicine [35] and as adapted to HIV/AIDS programs [36]. This will be accomplished by conducting an economic analysis from the payer and societal perspectives to estimate the cost, cost-effectiveness, and cost-utility of the intervention relative to standard of care.

Comprehensive cost analysis will be conducted to assess the cost of developing and implementing the ePrEP intervention, using a microcosting approach to estimate net costs. Cost-effectiveness analyses will include calculating the incremental cost-effectiveness ratio for the cost per HIV infection averted compared with standard of care as follows: (Cost_Intervention_ – Cost_StandardofCare_) / (Infections averted_Intervention_ – Infections averted_StandardofCare_). The health effect will be defined as the projected reduction in HIV infections over time associated with adopting the intervention relative to standard of care. The base, standard of care, model will estimate the number of infections expected in the absence of the intervention and may vary under different assumptions of baseline and clinic-based PrEP coverage outside of the intervention [37]. In the cost-utility analysis, we will calculate the cost per quality-adjusted life year (QALY) saved. QALYs saved by averting an HIV infection will be up-to-date estimates from the literature.

#### Qualitative Assessment

In-depth interviews will be conducted after the completion of 12-month assessments with key participants to explore the experience of intervention participants (up to 10) and standard of care participants (up to 5) over time. Participants will be selected using purposive sampling methods, seeking to gain a diverse set of trajectories in PrEP care. Topics will include (1) barriers and facilitators to PrEP care, (2) problems with and benefits of ePrEP or standard of care, (3) ways to address problems and amplify success of ePrEP or standard of care, and (4) factors that influence successful persistence in or falloff from PrEP care.

#### Safety Data

Safety data will be collected throughout the study for reports of adverse events, social harms, and side effects. Reportable events will be captured through EDC. A safety monitoring committee (SMC) will convene biannually and receive data reports on a quarterly basis. The SMC will be tasked with stopping the study if the intervention proves to be significantly outperforming the standard of care, or if the inverse occurs. We will conduct 1 interim analysis comparing the efficacy of the intervention in the active arm with the control arm, with efficacy based on differences in trial success versus failure using the primary outcome definition (detectable TFV-DP at 12 months). If the intervention is significantly outperforming the standard of care, the study will seek to adjust study design and supplements to open the intervention arm. The SMC will monitor several other factors, including HIV seroconversion, changes in kidney function, behavioral disinhibition, medication adherence, and differential loss to follow-up.

#### Trial Registration, Ethics, Consent, and Institutional Board Approval

This study has been approved by the University of North Carolina Institutional Review Board (number 18-0107). Clinical trial best practice will be followed in accordance with National Institutes of Health (NIH) guidance. The trial has been registered with ClinicalTrials.gov, trial number NCT03729570. Informed consent will be obtained before any study procedures are initiated.

## Results

We anticipate that the study will begin enrolling in May 2019. Our targeted enrollment period is 12 to 18 months. Therefore, we anticipate the completion of study data collection in mid to late 2021 and study results being available in 2022.

## Discussion

PrEP is highly effective in preventing HIV transmission. Similar to the predominant mode of biomedical prevention preceding it (condoms) [38], the most frequent failure is because of nonuse rather than the failure of the intervention itself [39]. The challenge for PrEP is therefore how to promote uptake and persistence in care among those groups at highest risk of HIV transmission. The delivery of PrEP services at a distance (and without the need for a provider visit) has the potential to overcome not only the geographic and transit-related barriers to care, but also the reluctance to seek care because of documented barriers to care such as stigma [40]. Pilot data from other studies indicate a strong interest in home-based PrEP service access [18] and survey data indicate an overall interest in PrEP [41]. Yet, there may be some disadvantages of PrEP home care. For instance, individuals may not develop strong bonds with clinicians via video, potentially leading to higher levels of discontinuation than standard care. This clinical trial will allow for the determination of whether telemedicine care increases or decreases initiation of and maintenance on PrEP. Provided that the ePrEP system provides gains relative to standard care, it will be important to develop strategies to sustain the program. The CDC’s high impact HIV prevention program [42] currently supports several mobile phone apps, and similar investment will be needed to support the coming wave of technology-based interventions such as ePrEP. Cost-effectiveness analyses, such as that proposed for this study, should inform health system investments.

As electronic, mobile phone-based communications emerge to be the predominant form of social interaction among youth [43], it is only natural that the health care systems reflect this change. The custom PrEP provision platform of the study will allow for an exploration of how youth can be retained in preventive care through an app-based telemedicine system. The recruitment of YMSM, with a focus on black and Latino participation, will provide an important perspective regarding how these highly impacted groups perform in telemedicine care relative to standard care. Provided that the study finds advantages of telemedicine, future work will be needed to understand how to best incorporate such a customized intervention into the broader health care system.

## Appendix

Multimedia Appendix 1 Electronic pre-exposure prophylaxis (ePrEP) study consent form.

Multimedia Appendix 2 Electronic pre-exposure prophylaxis (ePrEP) study baseline form.

## Abbreviations

ABMH: Andersen behavioral model adapted to HIV care
ATN: Adolescent Medicine Trials Network for HIV/AIDS Interventions
CDC: Centers for Disease Control and Prevention
DBS: dried blood spot
EDC: electronic data capture
ePrEP: electronic pre-exposure prophylaxis
FDA: Food and Drug Administration
FTC: emtricitabine
HIPAA: Health Insurance Portability and Accountability Act
MSM: men who have sex with men
NHBS: National HIV Behavioral Surveillance
NIH: National Institutes of Health
PrEP: pre-exposure prophylaxis
QALY: quality-adjusted life year
SMC: safety monitoring committee
SSC: specimen self-collection
STI: sexually transmitted infections
TDF: tenofovir disoproxil fumarate
TFV-DP: tenofovir diphosphate
YMSM: young men who have sex with men
