# Supplementary material for: An Electronic Pre-Exposure Prophylaxis Initiation and Maintenance Home Care System for Nonurban Young Men Who Have Sex With Men: Protocol for a Randomized Controlled Trial
Source: JMIR Res Protoc. 2019 Jun 10;8(6):e13982. doi: 10.2196/13982 (PMC6592500; doi:10.2196/13982)

# eprep RCT

**Emory University**

**On-line Consent to Participate in a Research Study**

**Main study consent**

**Participants age 18-24 years**

________________________________________________________________________

**UNC IRB Study # 18-0107**

**Consent Form Version Date:** Version 1.2 dated January 7, 2019

**Title of Study: ePrEP: Testing an electronic PrEP initiation and maintenance home care system to promote PrEP among adolescent MSM in rural and small town areas**

**Principal Investigator:** Aaron Siegler, PhD, MHS and Leandro Mena, MD MPH

**Principal Investigator Phone number:** 404-712-9733 and 601-984-5560

**Principal Investigator Email Address:** [asiegle@emory.edu](mailto:asiegle@emory.edu) and [lmena@umnc.edu](mailto:lmena@umnc.edu)

**Georgia Site Investigator:** Dr. Aaron Siegler, (404) 712-9733, [asiegle@emory.edu](mailto:asiegle@emory.edu)

**Mississippi Site Investigator:** Dr. James Brock, (601) 984-5560, [jbbrock@umc.edu](mailto:jbbrock@umc.edu)

**North Carolina Site Investigator:** Dr. Christopher Hurt, (919) 966-2789, [christopher_hurt@med.unc.edu](mailto:christopher_hurt@med.unc.edu)

**Sponsor:** The University of North Carolina at Chapel Hill (UNC-CH)

**Funding Source:** National Institutes of Health (NIH)

**Study Contact:** Karen Dominguez**,** MPH

**Study Contact telephone number:** 470-773-9704

**Study Contact email:** contact@eprepstudy.org

_________________________________________________________________

**What are some general things you should know about research studies?**

You are being asked to take part in a medical research study*.* To join the study is voluntary. You may refuse to join, or you may withdraw your consent to be in the study at any time, for any reason, without penalty. **It is entirely your choice. If you decide to take part, you can change your mind later on and withdraw from the research study.**

Research studies are designed to obtain new knowledge. This new information may help people in the future. You may not receive any direct benefit from being in the research study. There also may be risks to being in research studies.

Details about this study are discussed below. It is important that you understand this information so that you can make an informed choice about being in this research study. You will be given a copy of this consent form. You can ask the researchers named above, or study staff members who may assist them, any questions you have about this study at any time.

**What is the purpose of this study?**

The purpose of this study is to understand the acceptability of initiating and maintaining pre-exposure prophylaxis (PrEP) for HIV prevention through a home care system, called ePrEP. This involves having all the medical specimen collections, doctor visits, and medical assessments for PrEP to be done in your home rather than at a doctor’s office. This study is part of a series of studies called the iTEch network, which seeks to understand the use of technology to improve access to HIV prevention and care.

This study will include two groups of study participants: those who will have all study visits done at their home, and those who will visit a clinic for study visits.

Pre-exposure prophylaxis, or PrEP, is a way for people who do not have HIV but who are at risk of getting it to *prevent*HIV infection by taking a pill every day. The pill (brand name Truvada) contains two medicines (tenofovir and emtricitabine) can stop HIV from taking hold and spreading throughout your body. It is highly effective for preventing HIV if used as prescribed, but it is much less effective when not taken consistently. The medication is not free, but may be covered by health insurance or patient assistance programs.

**Are there any reasons you should not be in this study?**

You should not be in this study if any of the following apply to you:

- You are under the age of 18 or older than 24 years old
- You have been diagnosed with HIV
- You don’t understand, read, and speak English
- You are a hemophiliac
- You do not have a smartphone
- You are not willing to provide contact information for two family or friends that we can contact in case we are not able to get in touch with you

There may be other reasons you are not able to participate in this study.

**How many people will take part in this study?**

If you decide to participate, you will be one of 240 people in this study.

**How long will your part in this study last?**

Your participation will be required for 12 months.

**What will happen if you take part in the study?**

If you choose to be in the study, you will be asked to sign this consent form before you begin the study.

After you have provided your consent, you will give the study staff a list of ways to contact you, such as your e-mail address and phone number, as well as the phone numbers of two relatives or friends who know how to get in contact with you. Study staff will not leave phone messages unless you give permission. The study staff will also not tell your relative or friend anything about this study, your participation in the study, or give any information about you unless you give permission. Your contact information will be used to remind you to come in for your study visit and communicate with you about study procedures and results. You can also choose not to give any information that you do not want to give.

To participate in this study, you will need to download an app called eP to your smart phone. After downloading the app, you will complete an initial electronic survey. The survey will ask detailed questions about your sexual history, medical history and substance use history. A home specimen collection kit will be mailed to you. The kit will come in a box that will contain instructions and materials to collect your own blood, urine, rectal swab, and throat swab samples.

Study staff will also verify you have been vaccinated for hepatitis B by checking the state vaccination records and will ask you to be tested to see if you have Hepatitis B. The options for this testing are as follows:

1. You can go to the nearest LabCorp or Quest center and have blood drawn for a hepatitis B test. The cost will be covered by the study; OR
2. You can use the home specimen kit provided for the study (described above) and do a second fingerprick to collect blood for a hepatitis B test, and take this to a local lab for testing; OR
3. If the test is offered at your local health department, you can have a hepatitis B test done at no cost.

After you complete this step and mail back the self-collected specimens, study staff will confirm that you are eligible to participate. If eligible to continue with the study, you will be randomized. Randomization means you will be “randomly” assigned by a computer to one of two groups. Half of the participants will be in the group that completes home specimen collection kits, electronic surveys, and telemedicine visits (remote care). The other half will be in the group that is linked to a local PrEP provider in your area for in-person visits and labs. You will have to schedule a visit with a PrEP clinic in your area, this could be at a health department or specialty clinic. The lab tests and prescriptions will be managed through this clinic. You will not be able to choose which group you are assigned to.

*Remote care:* If you are randomized to remote care, you will have a “virtual” doctor’s visit, using video-chat or telephone to talk with a study doctor via the eP app. If after the virtual visit you are still interested in and eligible to receive a PrEP prescription, a study doctor will send your PrEP prescription to the pharmacy of your choice. Collecting home specimens will take approximately an hour. Initial surveys will take about 45 minutes to complete. The virtual visit with your study clinician will take 20-30 minutes.

*In-person visits:* If you are randomized to see your provider in-person, study staff will assist in linking you to the closest provider who can offer you the treatment to decrease your risk of getting HIV (called a “PrEP provider”). Your local provider visits, PrEP prescriptions, and labs will be in-person and not through the study. Your visits costs will not be covered by the study. You will complete electronic surveys via the eP app. At 3, 6, 9, and 12 months you will be asked to complete a follow-up survey. Each survey will take approximately 45 minutes. At 6 months and 12 months you will be sent a Dried Blood Spot card in the mail. You will be asked to complete the specimen collection and send it back. You will not have to pay for this. Completing the dried blood spot (DBS) specimen collection will take approximately 30 minutes. (Described further in Home Specimen Collection below.)

One month after receiving and filling a PrEP prescription, you will be asked to talk with study staff about common issues regarding PrEP. Using the eP app on your phone, you will have a videochat with a study counselor or a study doctor. This is expected to take no more than 30 minutes.

You will have an additional follow-up two months later (3 months after PrEP prescription). You will complete a home specimen collection, sexual behavior questions, electronic survey, and videochat with the study doctor. You will complete this process every 3 months for 12 months. The 3, 6, and 9 month assessment are expected to take approximately 1.5 hours in total.

At the 12 month follow-up, you will complete the final study assessment. You will complete another set of home specimen collection, sexual behavior questions, electronic survey, and a videochat with the study doctor. There will be a final survey about your experiences with the ePrEP system. This will be the end of your participation in the study. If you would like to continue taking PrEP, study staff will provide you with information on how to continue your prescription. This final assessment is expected to take approximately 1.5 hours in total.

*Home Specimen Collection:* Using the home specimen kit will include collecting a rectal swab sample, a throat swab sample, a urine sample, and finger prick blood samples. Rectal swab collection will involve inserting the tip of a swab about one and a half inches to collect a sample. Throat swab collection will involve using a swab to reach the rear area of your throat, collecting a sample with the swab tip. Urine sample collection will involve urinating in a cup and transferring urine with a pipette from the cup to the sample tube. You will receive detailed printed instructions regarding how to collect each of these samples.

You will also receive printed and video instructions in self-administered finger prick blood draw methods. You will conduct 1-2 self-administered finger pricks, similar to the practice someone with diabetes might follow on a regular basis. Although this is a smaller needle than used for a traditional blood draw, you may experience some pain from it. The finger prick device is spring-loaded and encased in a plastic shell, so you will not see or manipulate the needle. Following the finger pricks, you will collect blood by blotting your finger on collection paper (for DBS) and by using a collection tube. This will be a small amount of blood, about 6 drops total. If the sight of blood makes you feel light-headed, or if at any point you feel uncomfortable or wish to stop participating, please immediately notify study staff. You will be provided with a call-in line to receive help with any unexpected problems.

*Labs:* After collecting the samples, you will mail them to Emory University in a prepaid mailer included in the kit. Your samples will be used to conduct tests typical for a PrEP visit. This includes sexually transmitted infection (STI) testing for gonorrhea, chlamydia, syphilis, and HIV. Under state law, confidentiality does not extend to certain communicable diseases, such as tuberculosis (TB), HIV, hepatitis, or other illnesses that put others at risk. If the researchers become aware that subjects have such an illness, they are required to report it to state authorities.

Laboratory tests will also be done to check your kidney function. We will share these results with the study doctor to inform your “virtual” visit. We will not sell your samples. Your test results will be returned to you by study staff or the study doctor. At 6 and 12 months you will be asked to complete a Dried Blood Spot (DBS) card to test PrEP drug levels in your system. The DBS card is for research purposes and the results will not be returned to you. If you agree, your samples and health information will be stored and available to help answer research questions, such as research to understand certain diseases, or to develop new scientific methods.

Our syphilis test is performed with a standard syphilis test and determines a preliminary positive result. It does not provide detailed results like a similar test from a doctor’s office or health department. Therefore, if you test preliminary positive for syphilis, we will recommend that you seek additional testing from either your doctor or health department.

*Surveys:* You will also be asked to complete an electronic survey with questions similar to those doctors usually ask of PrEP patients. This will be done using the app. You will also be asked about your HIV knowledge, sexual behavior, stigma and social support. Your answers to these questions will be shared with the study doctor. There will be a section of the survey that includes questions about your experience using ePrEP and other research.

You may also be asked to participate in an in-depth interview. If chosen, you will be asked to consent separately for the interview. The purpose of the interview will be to collect more information on your experiences as a participant on the study, the app, and PrEP use.

Since PrEP is recommended in combination with safer sex practices, you will also be encouraged to practice condom-protected sex throughout the duration of your participation in the study and at all times that you are on PrEP.

**How will my medicine be provided?**

The medicine that you will take will be dispensed by the pharmacy of your choice. If you have questions about taking PrEP, you should ask the principal investigator, study doctor or study staff. You may also call the pharmacy if you have questions about the medicine.

**What are the possible benefits from being in this study?**

You will be tested for HIV and STIs and be linked to treatment if necessary. You will have a lower chance of acquiring HIV if you take PrEP as directed. The study results may be used to help others in the future, if the information we learn helps improve HIV and STI prevention services.

**What are the possible risks or discomforts involved from being in this study?**

As with all research, there are risks associated with this study. You may experience physical discomfort from the self-administered specimen collection, including the finger prick, rectal swab, and throat swab. You may also experience bruising around the site of the finger prick. You may find out that you are infected with HIV or another STI. This might upset you. If you are infected with HIV or another STI, we will help you find a clinic to follow-up for treatment. For HIV, we will also provide you with additional lab tests to help you get into care.

Some of the questions in the surveys are personal, and may make you uncomfortable. We hope you will answer all questions to the best of your ability. You can choose not to answer any question that makes you uncomfortable. We will keep information about your HIV and STI testing, and your responses to the survey questions in a secure database, only study staff will have access to the secure database. Although we will take steps to reduce the chance, there is a small chance that someone other than study staff might see your study information. More information about how we will protect your confidentiality is below.

The most common risks and discomforts associated with PrEP are: nausea, headache, and flatulence. These side effects are uncommon and usually resolved within the first month of taking PrEP. The less common risks and discomforts associated with PrEP are: decrease in bone mineral density, moderately decreased kidney function. Rare but possible risks include: acute kidney injury. We will monitor your creatinine levels to decrease any potential risks related to renal function.

We will make every effort to protect your confidentiality. Your study ID number will only be linked to your name and contact information within the study’s secure, HIPAA compliant enrollment database. This database will not be linked to any of your other study records or data you provide during the study. Only approved research staff members at Emory University, University of Mississippi Medical Center and University of North Carolina-Chapel Hill can access this database using a secure server, password, and cell phone confirmation. All of your study records and study data are stored in a separate, unlinked HIPAA compliant database. Research staff members involved in this study are required to sign a form stating that they will protect and keep private all information on every person in the study.

It is possible that the researchers will learn something new during the study about the risks of being in it. If this happens, they will tell you about it. Then you can decide if you want to continue to be in this study or not. You may be asked to sign a new consent form that includes the new information if you decide to stay in the study.

**What if you want to stop before your part in the study is complete?**

You can withdraw from this study at any time, without penalty. If you leave the study before the final planned study visit, the researchers may follow up with you on completing final study procedures. The investigators also have the right to stop your participation at any time. This could be because you have failed to follow instructions, have undermined the right or privacy of another participant, or because the entire study has been stopped.

**How will your privacy be protected?**

Your participation in this study will be kept confidential and private as permitted by law. This includes the information you provide during the discussion today.

Every effort will be taken to protect your identity as a participant in this study. You will not be identified by name in any report or publication of this study or its results. Instead, you will be known only through a study ID number. Any data linking your name to your study ID number will be kept in a locked cabinet in a locked room at the study site, but separate from where your study records are stored. Most of your study records will be kept electronically on secure databases with password required login. Staff members involved in this study are required to sign a form stating that they will protect and keep private all information on every person in the study.

No subjects will be identified in any report or publication about this study. Although every effort will be made to keep research records private, there may be times when federal or state law requires the disclosure of such records, including personal information. This is very unlikely, but if disclosure is ever required, Emory University will take steps allowable by law to protect the privacy of personal information. In some cases, your information in this research study could be reviewed by representatives of Emory University, University of Mississippi Medical Center, the University of North Carolina at Chapel Hill (UNC-CH), research sponsors, or government agencies for purposes such as quality control or safety.

At the end of the study, all of your information from the study will be de-identified and stored at Emory University in Atlanta, Georgia.

A description of this study will be available on http://clinicaltrials.gov, as required by U.S. Law. This website will not include information that can identify you. At most, the website will include a summary of the results. You can access this website at any time.

To help further protect your privacy, the Adolescent Trials Network (ATN) has obtained a Certificate of Confidentiality from the U.S. Department of Health and Human Services (DHHS). It adds special protection for research information that identifies you. It says that we do not have to identify you, even under a court order or subpoena. With this Certificate, the researchers cannot be forced to disclose information that may identify you, even by a court subpoena, in any federal, state, or local civil, criminal, administrative, legislative, or other proceedings. The researchers will use the Certificate to resist any demands for information that would identify you, except as explained below.

The Certificate cannot be used to resist a demand for information from personnel of the United States Government that is used for auditing or evaluation of Federally funded projects or for information that must be disclosed in order to meet the requirements of the federal Food and Drug Administration (FDA).

Still, we may report medical information (if you need medical help), probable harm to yourself or others, and the government may see your information if it audits us. This Certificate does not mean the government approves or disapproves of our project. You should understand that a Certificate of Confidentiality does not prevent you or a member of your family from voluntarily releasing information about yourself or your involvement in this research. If an insurer, employer, or other person obtains your written consent to receive research information, then the researchers may not use the Certificate to withhold that information.

Every effort will be made to keep your participation in the study and any personal information about you private and confidential. However, absolute confidentiality cannot be guaranteed. For example, if a study staff member learns something that would immediately put you or others in danger, the study staff member is required by law to take steps to keep you and others safe. This means that study staff members have to report to the authorities (hospital, police, or social services) any information you say that suggests that you might be in danger, such as telling study staff that you plan to hurt or kill yourself, hurt or kill someone else, or if someone is abusing or neglecting you.

In addition, your records may be reviewed by certain agencies or people who make sure that the study staff are doing what they are supposed to and everyone in the study is being protected. Under the guidelines of the Federal Privacy Act, the sponsoring agency at the National Institutes of Health (NIH) and the UNC IRB may look at your records. If your study records are reviewed, your identity could become known to them. However, these persons are expected to maintain your individual confidentiality. This means that they will not tell others information about you or that you are in the study. By signing this form, you are allowing such access.

If you live in the state of Mississippi and receive care at the University of Mississippi Medical Center, a copy of this informed consent will be included in your health record. Individuals involved in your treatment, who obtain information for payment of services, or who access information for health care operations may have access to your research records.

**Will you receive anything for being in this study?**

The study will compensate all participants for the initial study visit with $50. Compensation for follow-up visits will be $40 for electronic survey completion (months 3,6,9 and 12) and $40 for dried blood spot collection (at months 6 and 12). Compensation will be distributed to you via a reloadable debit card that will be reloaded at the completion of each visit.

**Are there any costs to you for taking part in this study?**

Participation: There will be no costs to you for participating in this study, other than basic expenses like transportation to a FedEx drop box for mailing specimens. You will not be charged for any of the research activities, including the postage costs of mailing the specimens you provide.

PrEP medication: The cost of PrEP medication will not be covered by this study. If you have health insurance, we will assist you in accessing PrEP coverage through your insurance and also accessing copayment assistance programs. If you do not have health insurance, we will help you enroll in drug access assistance programs or, if you are interested, help you enroll in health insurance. However, the study does not pay for medication costs that are not covered by insurance or assistance programs. We will work with you to make sure you are aware of any out-of-pocket costs before accessing PrEP.

## **In Case of Injury**

If you live in the state of Georgia: If you get ill or injured from being in the study, Emory will help you get medical treatment. Emory and the sponsor have not, however, set aside any money to pay you or to pay for this medical treatment. The only exception is if it is proven that your injury or illness is directly caused by the negligence of an Emory or sponsor employee. “Negligence” is the failure to follow a standard duty of care. If you become ill or injured from being in this study, your insurer will be billed for your treatment costs. If you do not have insurance, or if your insurer does not pay, then you will have to pay these costs. If you believe you have become ill or injured from this research, you should contact Dr. Siegler at 404-712-9733. You should also let any health care provider who treats you know that you are in a research study.

If you live in the state of North Carolina: All research involves a chance that something bad might happen to you.  If you are hurt, become sick, or develop a reaction from something that was done as part of this study, the researcher will help you get medical care, but the University of North Carolina at Chapel Hill has not set aside funds to pay you for any such injuries, illnesses or reactions, or for the related medical care.

If you live in the state of Mississippi: In the case of injury or illness resulting from your participation in this study, medical treatment is available to you at the University of Mississippi Medical Center. You will be charged the usual and customary charges for any such treatment you receive.

**Who is sponsoring this study?**

This research is being sponsored by the University of North Carolina at Chapel Hill and funded by the National Institutes of Health (NIH). This means that the sponsor, the University of North Carolina at Chapel Hill (UNC-CH), is providing money from NIH to Emory University and the University of Mississippi Medical Center to help conduct this study. The researchers do not, however, have a direct financial interest with the sponsor or funding source or in the final results of the study.

**What if you have questions about this study?**

You have the right to ask, and have answered, any questions you may have about this research at any time before, during, or after your participation. The principal investigators in charge of this study are Dr. Aaron Siegler or Dr. Leandro Mena. The site investigator for Mississippi is Dr. James Brock, (601) 984-5560. The site investigator for North Carolina is Dr. Christopher Hurt, (919) 966-2789. The site investigator for Georgia is Dr. Aaron Siegler, (404) 712-9733. The Study Coordinator is Karen Dominguez. If you ever have questions, or concerns about this study, you may call Karen Dominguez at 404-727-9788 or on the study phone at 470-773-9704.

**What if you have questions about your rights as a research participant?**

All research on human volunteers is reviewed by a committee that works to protect your rights and welfare. If you have questions or concerns about your rights as a research subject you may contact, anonymously if you wish, the University of North Carolina at Chapel Hill (UNC-CH) Institutional Review Board at 919-966-3113 or by email to IRB_subjects@unc.edu.

**Optional Future Research**

Research with blood specimens can help researchers understand how the human body works. Research can also answer other questions by using specimens. Sometimes researchers collect and store many specimens together and use them for different kinds of research, or share them with other scientists; this is called a specimen repository or "biobank."

With your consent, the blood samples you send to Emory will be stored in a biobank for future research. The samples will be stored in a laboratory to which only limited staff have access. The samples will not be stored with any personal identifying information. The samples will be coded and only the researchers at Emory will have access to the file that could identify the samples as belonging to you. The samples could be kept for an indefinite period of time. Other researchers at Emory or other institutions may use the samples only if they first gain permission from the researchers involved in the ePrEP study. Research studies may be done at many places at the same time. Your personal identifying information will not be sent to other researchers.

There are no benefits to you from research conducted using your samples. There is a risk of breach of confidentiality.

There is no cost to you for storage and use of the specimens for research purposes. You will not receive anything for the use of your specimens.

Any samples obtained for this purpose become the exclusive property of the researchers working on the iTech research program.

I voluntarily agree that my blood and information can be stored. I understand it may be used in

future research to learn about, prevent, or treat health problems.

In addition, I have made the optional choice marked below. I know that I can take still take part in the study, even if I answer ‘no’ to this option.

- Yes, I give permission to have my samples stored for future use.
- No, I do NOT give permission to have my samples stored for future use.

**UNC IRB Study # 18-0107**

**Title of Study: ePrEP: Testing an electronic PrEP initiation and maintenance home care system to promote PrEP among rural, adolescent MSM**

**Principal Investigators:** Aaron Siegler, PhD and Leandro Mena, MD MPH

**Participant’s Agreement (for online consent):**

If you agree to voluntarily participate in this study, please select the appropriate response. You may print a copy of this consent form for your own records.

- I agree to participate in this research study. (1)
- I do NOT agree to participate in this research study. (2)


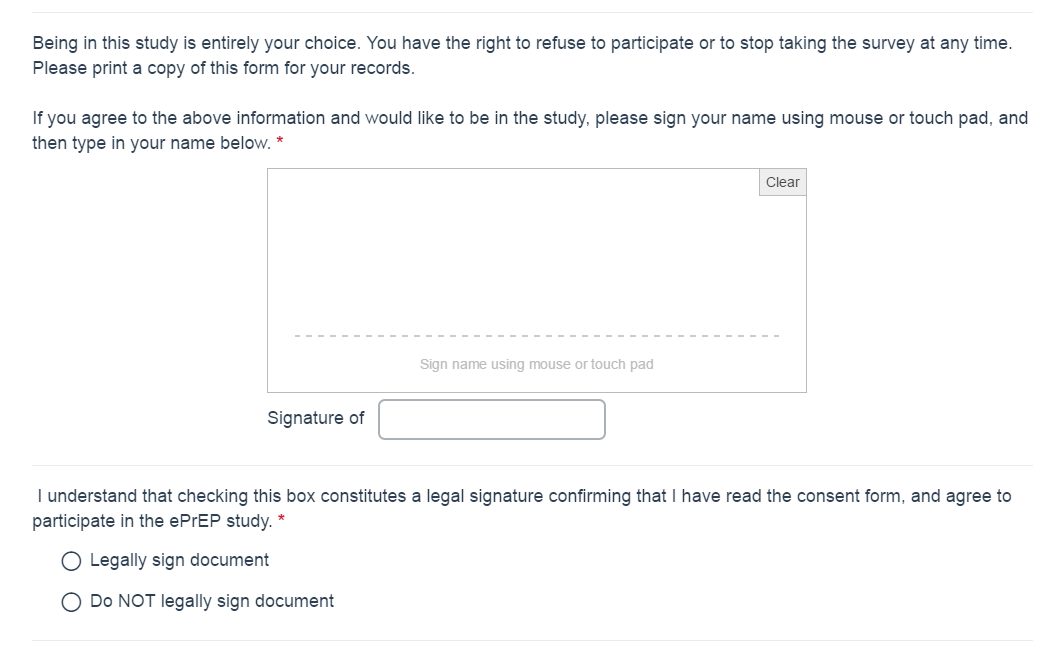

Supplement: Multimedia Appendix 1 [file resprot_v8i6e13982_app1.docx]
