# Supplementary material for: An Electronic Pre-Exposure Prophylaxis Initiation and Maintenance Home Care System for Nonurban Young Men Who Have Sex With Men: Protocol for a Randomized Controlled Trial
Source: JMIR Res Protoc. 2019 Jun 10;8(6):e13982. doi: 10.2196/13982 (PMC6592500; doi:10.2196/13982)
Supplement: Multimedia Appendix 2 [file resprot_v8i6e13982_app2.docx]

**ePrEP Baseline Survey**

Contents

[Demographics 2](#_Toc519765678)

[Marital Status 3](#_Toc519765679)

[Acute Symptoms 4](#_Toc519765680)

[HIV Negative Cascade (ATN Standard Domain- FINAL) 5](#_Toc519765681)

[Sexual History 9](#_Toc519765682)

[Mental Health- iTech specific: 11](#_Toc519765683)

[Resiliency 13](#_Toc519765684)

[Geospatial (using map) 14](#_Toc519765685)

[Basic HIV knowledge 15](#_Toc519765686)

[PrEP Intentions 15](#_Toc519765687)

[PrEP Stigma 16](#_Toc519765688)

[Other sexual risk items specific to iTech: 16](#_Toc519765689)

[Sexual Stigma 17](#_Toc519765690)

[Perceived HIV risk (From SHIPP) 17](#_Toc519765691)

[PrEP Use Self-Efficacy 18](#_Toc519765692)

[Internalized Homophobia 19](#_Toc519765693)

[Health Literacy 20](#_Toc519765694)

[Outness to Primary Medical Care Provider 21](#_Toc519765695)

[Substance Use/Abuse 22](#_Toc519765696)

[Injection Behaviors 32](#_Toc519765697)

[Religiosity 32](#_Toc519765698)

[Technology Use Questions 33](#_Toc519765699)

**Module 1**

# Demographics

1. What is your date of birth? (enter month, day, year) (ATN)
   1. MMDDYYYY
2. Do you consider yourself Hispanic or Latino? (ATN)
   1. Yes
   2. No
3. What race are you? (ATN)
   1. Asian/Pacific Islander
   2. Black/African-American
   3. White/Caucasian
   4. Native American/ Alaska Native
   5. Mixed race
   6. Other
4. [Logic: if answers “e” above] You indicated that you are mixed race. Which term best describes you?
   1. Asian/Pacific Islander
   2. Black/African-American
   3. White/Caucasian
   4. Native American/Alaska Native
   5. Other
5. What is the 5-digit ZIP code for the location where you primarily live? (ATN)
6. What is your current gender identity? (ATN)
   1. Female
   2. Male
   3. Trans female or trans woman
   4. Trans male or trans man
   5. Genderqueer or gender nonconforming
   6. Other, specify_____
   7. Decline to answer
7. What sex were you assigned at birth, on your original birth certificate? (ATN)
   1. Female
   2. Male
   3. Decline to answer
8. What is your current sexual identity? (ATN)
   1. Bisexual
   2. Gay, queer, same gender loving or homosexual
   3. Straight or heterosexual
   4. Other, specify___
   5. Decline to answer
9. What is the highest level in school that you completed?
   1. College, post graduate, or professional school
   2. Some college, Associate’s Degree and/or Technical School
   3. High school or GED
   4. Did not finish high school
10. What best describes your employment status? Are you: (Check all that apply)
    1. Employed full-time
    2. Employed part-time
    3. A full-time student
    4. A part-time student
    5. On active duty in US Armed Forces, Reserves, or National Guard
    6. Unable to work for health reasons
    7. Unemployed
    8. Other Please enter an ‘other’ value for this section
11. What was your household income last year from all sources before taxes? (month/yearly)
    1. $0 to $417 (monthly)/$0 to $4,999 (yearly)
    2. $418 to $833 (monthly) / $5,000 to $9,999 (yearly)
    3. $834 to $1250 (monthly) / $10,000 to $14,999 (yearly)
    4. $1251 to $1999 (monthly) / $15,000 to $23,999 (yearly)
    5. $2000 to $2500 (monthly) / $24,000 to $29,999 (yearly)
    6. $2501 to $3333 (monthly) / $30,000 to $39,999 (yearly)
    7. $3334 to $4167 (monthly) / $40,000 to $49,999 (yearly)
    8. $4168 to $4999 (monthly) / $50,000 to $59,999 (yearly)
    9. $5000 to $6250 (monthly) / $60,000 to $74,999 (yearly)
    10. $6251 or more (monthly) / $75,000 or more (yearly)
    11. Don’t know
    12. Decline to answer
12. Where does your current health insurance coverage come from? If you are covered by more than one health insurance plan, please tell us about your PRIMARY plan, the one that you mainly use.
    1. Through my employer
    2. An Affordable Care Act (ACA) or “Obamacare” plan
    3. My parent’s insurance
    4. Medicaid
    5. Medicare
    6. TRICARE (CHAMPUS)
    7. Veterans Administration (VA)
    8. Other, please specify: __________________
    9. Don’t know
    10. Don’t have health insurance
    11. Decline to answer

# Marital Status

1. How do you define your primary relationship status? (adjusted ATN other)
   1. I am single
   2. I am casually dating
   3. I have a boyfriend or partner
   4. I am in a civil union or domestic partnership
   5. I am legally married
   6. Decline to answer
2. Is/was this partner: [Logic: If answer to 13 is c, d,e,]
   1. Male
   2. Female
   3. Transgender female or transwoman
   4. Transgender male or transman
   5. Decline to answer
3. How do you and your partner handle sex outside of your relationship? (If response other than single or casually dating) (ATN other)[Logic: If answer to 13 is c, d, e]
   1. Neither of us has sex with others; we are monogamous
   2. Only I have sex with others
   3. Only my partner has sex with others
   4. Both us have sex with others separately
   5. Both of us have sex with others together
   6. We both have sex with others separately and together
   7. I have sex with others, but don’t know about my partner
   8. I don’t have sex with others, but I don’t know about my partner
   9. Decline to answer

# Acute Symptoms

1. Have you experienced any of the following symptoms in the **last month**? Check all that apply.
   1. Fever
   2. Sore throat
   3. Swollen glands (lymph nodes)
   4. Skin rash
   5. Fatigue
   6. Night sweats
   7. Unintentional weight loss
   8. Body or joint aches
   9. Headache
   10. Nausea and/or vomiting
   11. Diarrhea
   12. Sores in your mouth
   13. I haven’t experienced any of these symptoms
2. For your medical care as part of this study, we need to know your weight. Please be as accurate as possible. How much do you weigh? _____ pounds
3. Have you ever been told by a doctor that you have any of the following? (Check all that apply)
   1. High blood pressure
   2. Diabetes
   3. Kidney disease
   4. Hepatitis B
   5. Recent blood clot or put on blood thinners
   6. None of the above

# HIV Negative Cascade (ATN Standard Domain- FINAL)

| **HIV Negative Cascade Characteristics** | | |
| --- | --- | --- |
| **Characteristic** | **Format/Values** | **Comments** |
|  |  |  |
| **Health Care**  Have you visited a doctor, nurse, or other health care provider in the past 12 months? | Coded Fields:   1. Yes 2. No   Decline to answer |  |
| **Primary Health Care Provider**  Do you have a primary health care provider (for example, a pediatrician, a doctor, or other health care provider that you see regularly)? | Coded Fields:   1. Yes 2. No   Decline to answer |  |
| ***Ask if Primary Health Provider*** *is* ***Yes***  Are you comfortable discussing your sexual relationships with your primary health care provider? | 1. Yes 2. No   Decline to answer |  |
| **Lifetime HIV Testing**  Have you ever had an HIV test? | Coded Fields:   1. Yes 2. No   Decline to answer | **Source:** NHBS |
| *Ask if* ***Lifetime HIV Testing*** *is* ***Yes***  **First HIV Test Date**  When did you have your first HIV test? If you do not remember please give your best estimate.  *Please enter the month and year.* | MMYYYY  Decline to answer | **Source:** NHBS |
| *Ask if* ***Lifetime HIV Testing*** *is* ***Yes***  **Ever Tested HIV Positive**  Have you ever tested positive for HIV? (Do you have HIV?)  *This includes having gotten the virus earlier, but are now suppressed (undetectable).*  *If yes, alert study staff | Coded Fields:   1. Yes 2. No   Decline to answer | **Source:** NHBS |
| *Ask if* ***Lifetime HIV Testing*** *is* ***Yes***  **Most Recent HIV Test Date**  When did you have your most recent HIV test?  *Please enter the month and year.* | MMYYYY  Decline to answer | **Source:** NHBS  Include logic check: Date should not be earlier than First HIV Test Date. |
| *Ask if* ***Lifetime HIV Testing*** *is* ***Yes***  **Most Recent Test Result**  What was the result of your most recent HIV test? | Coded Fields:   1. Negative, you do NOT have HIV 2. ~~Positive, you DO have HIV~~ 3. Indeterminate 4. Never obtained results 5. Have not yet been provided results   Decline to answer | **Source:** NHBS |
| **Lifetime STI Testing**  A sexually transmitted infection (STI) is an infection transmitted through sexual activity such as syphilis, gonorrhea, chlamydia, herpes, or genital warts.  Have you ever been tested for an STI that was not HIV? | Coded Fields:   1. Yes 2. No   Decline to answer | **Source:** Element |
| **STI Diagnosis 6 months**  Please indicate whether you have been diagnosed with any of the following sexually transmitted infections in the past 6 months  *Please select all that apply.* | Coded Fields:   1. Chlamydia 2. Genital warts, anal warts, HPV 3. Gonorrhea 4. Hepatitis B 5. Hepatitis C 6. Herpes, HSV1/HSV2 7. Syphilis 8. Urethritis   Decline to answer | **Source:** Scale It Up |
| **Circumcision**  Is your penis circumcised (cut) or uncircumcised (uncut)? | 1. Circumcised (cut)  2. Uncircumcised (uncut)  3. Don’t know  Decline to answer | Source: Involvement |
| **Post-Exposure Prophylaxis** **(PEP) Awareness**  Before today, have you ever heard of people taking anti-HIV medicines AFTER a sexual or drug use exposure, to reduce the risk of getting HIV? This is called post-exposure prophylaxis, or PEP. | Coded Field:   1. Yes 2. No   Decline to answer | **Source:** UCSF Risk Assessment |
| Ask if **PEP Awareness** is **Yes**  **Lifetime PEP Use**  Have you ever taken post-exposure prophylaxis (PEP) AFTER a sexual or drug use exposure, to reduce the risk of getting HIV? | Coded Field:   1. Yes 2. No   Decline to answer | **Source:** UCSF Risk Assessment with wording changed to match NHBS PrEP question |
| *Ask if* ***Lifetime PEP Use*** *is* ***Yes***  **PEP Use 12 Months**  In the past 12 months, have you taken PEP (post-exposure prophylaxis) AFTER a sexual or drug use exposure, to reduce the risk of getting HIV? | Coded Field:   1. Yes 2. No   Decline to answer | **Source:** NHBS |
| VIDEO: [Whatisprep.org](file:///\\nasn2acts.cc.emory.edu\researchdata-ts\epiprojs\ePrEP%20RCT\Surveys\whatisprep.org) | | |
| **Pre-Exposure Prophylaxis** **(PrEP) Awareness**  Before being involved in this study, had you ever heard of people regularly taking anti-HIV medicines BEFORE a sexual or drug use exposure, to reduce the risk of getting HIV? This is called pre-exposure prophylaxis, or PrEP. | Coded Field:   1. Yes 2. No   Decline to answer | **Source:** NHBS |
| *If PrEP Awareness = Yes*  Where did you hear about PrEP? Check all that apply. | 1. A friend or family member 2. A sex partner 3. An HIV prevention counselor 4. On a website 5. At a community meeting 6. From a poster or flyer 7. Other, specify: | **Source:** UMMC |
| **Lifetime PrEP Use**  Have you ever taken PrEP?  *If yes, alert study staff | Coded Field:   1. Yes, I am on PrEP right now 2. Yes, I was in the past, but I’m not on PrEP anymore 3. No, I’ve never taken PrEP   Decline to answer | **Source:** Scale It Up - *Stage 4b: PrEP Action* |
| *Ask if* ***Lifetime PrEP Use*** *is* ***No, I have never take PrEP***  **Barriers to PrEP Uptake**  Which of the following are reasons that you have not previously started PrEP? ***Check all that apply*** | Coded Field:   1. I had not heard about PrEP until this study [skip to next section] 2. I didn’t know how to get the costs of PrEP covered 3. I didn’t know where to go to get PrEP 4. I tried to access PrEP, but my doctor wouldn’t prescribe it for me 5. I was concerned that PrEP might not provide complete protection against HIV 6. I was nervous about side effects that might make me sick 7. I was worried about the long-term effects of PrEP on my health 8. Other, specify ________   Decline to answer | **Source:** Adapted from previous questions for those who never used PrEP. |

# Sexual History

**In the next section, we will be asking about your sexual behaviors. Some of the questions may seem similar, but are asking about different time periods. Please be aware of the time period we are asking about before you answer the question. Also, if you are unsure of exact numbers, please answer with your best approximation.**

| **Sexual Behavior and Risk** | | |
| --- | --- | --- |
| **Characteristic** | **Format/Values** | **Comments** |
| How old were you when you had sexual intercourse for the first time with somebody else? | Integer  Decline to answer | Include logic check that response does not exceed current age |
| During your life, who have you had anal sex with?  Check all that apply. | Coded Field:  1. Females  2. Males  3. Transgender females  4. Transgender males  Decline to answer |  |
| In the **past 3 months**, who have you had sexual intercourse with?  Check all that apply | Coded Field:  1. Females  2. Males  3. Transgender females  4.Transgender males  Decline to answer |  |
| **The last time** you had sexual intercourse, did you drink alcohol or use drugs before having sex? | Coded Field:  1. Yes  2. No  Decline to answer |  |
| **No Condom Lifetime**  In your life, have you ever had anal sex WITHOUT using a condom? | Coded Field:   1. Yes 2. No   Decline to answer | **Source:** NHBS adapted to lifetime question |
| *Ask if* ***No Condom Lifetime*** *is* ***Yes***  **No Condom HIV Negative**  In the **past 3 months**, did you have anal sex WITHOUT a condom with a partner you knew was HIV negative? | Coded Field:   1. Yes 2. No   Decline to answer | **Source:** NHBS – changed time period to 3 months |
| *Ask if* ***No Condom Lifetime*** *is* ***Yes***  **No Condom HIV Positive**  In the **past 3 months**, did you have anal sex WITHOUT a condom with a partner you knew was HIV positive? | Coded Field:   1. Yes 2. No   Decline to answer | **Source:** NHBS – changed time period to 3 months |
| *Ask if* ***No Condom Lifetime*** *is* ***Yes***  **No Condom HIV Unknown**  In the **past 3 months**, did you have anal sex WITHOUT a condom with a partner whose HIV status you didn’t know? | Coded Field:   1. Yes 2. No   Decline to answer | **Source:** NHBS – changed time period to 3 months |
| Do you consider yourself to be a top, bottom, or versatile? | 1. Top 2. Bottom 3. Versatile 4. Decline to answer | **Source:** AMIS |
| In the **past 3 months**, with how many partners have you had anal sex? (include male or transgender female partners) | Integer | **Source:** adjusted from AMIS |
| **Verified partner HIV status**  For how many of these [# partners] have you done couples HIV testing with OR have seen medical documentation of their HIV test results? | Integer |  |
| What is the HIV status for the [# verified HIV status] partners whose HIV status you seen medically documented? | 1. HIV positive ___ 2. HIV negative___ | Logic: integers must total verified partner HIV status |
| How many of these [# verified HIV positive] HIV positive partners, do you know to be on antiretroviral therapy? | Integer | Logic: can’t be > number of verified HIV positive partners |
| **HIV positive on ART**  **In the last 3 months**, how many times did you have anal sex with these partners on antiretroviral therapy? | Integer |  |
| **Receptive Anal Sex**  Of [# of acts] times, how many were receptive anal sex (meaning you were the bottom)? | Integer | Logic: can’t be > number of acts |
| Of [# of receptive acts] receptive times, how many were fully protected by condoms? | Integer | Logic: ask if >0 receptive acts |
| **Insertive Anal Sex**  Of [# of acts - # of receptive acts] insertive acts, how many were fully protected by condoms? | Integer | Logic: Do not ask if total acts already accounted for |
| **HIV positive**  Now we are going to ask about the partners you do NOT know to be on antiretroviral therapy, which is [n] partners. **In the last 3 months**, how many times did you have anal sex with these partners? | Integer | Logic: n= HIV positive – known to be on ART |
| **Receptive Anal Sex**  Of [# of acts] times, how many were receptive anal sex (meaning you were the bottom)? | Integer | Logic: can’t be > number of acts |
| Of [# of receptive acts] receptive times, how many were fully protected by condoms? | Integer | Logic: ask if >0 receptive acts |
| **Insertive Anal Sex**  Of [# of acts - # of receptive acts] insertive acts, how many were fully protected by condoms? | Integer | Logic: Do not ask if total acts already accounted for |
| **HIV Unknown**  Now we are going to ask about the [# HIV unknown] partners for whom you have not seen a medically documented HIV status. . **In the last 3 months**, how many times did you have anal sex with these partners? | Integer |  |
| **Receptive Anal Sex**  Of [# of acts] times, how many were receptive anal sex (meaning you were the bottom)? | Integer | Logic: can’t be > number of acts |
| Of [# of receptive acts] receptive times, how many were fully protected by condoms? | Integer | Logic: ask if >0 receptive acts |
| **Insertive Anal Sex**  Of [# of acts - # of receptive acts] insertive acts, how many were fully protected by condoms? | Integer | Logic: Do not ask if total acts already accounted for |

# Mental Health- iTech specific:

**PHQ-2/GAD-2 screener**

Over the past 2 weeks, how often have you been bothered by any of the following problems?

|  | **Not at all**  **(0)** | **Several days**  **(1)** | **More than half the days**  **(2)** | **Nearly every day**  **(3)** | **Decline to answer** |
| --- | --- | --- | --- | --- | --- |
| 1. Little interest or pleasure in doing things | ( ) | ( ) | ( ) | ( ) | ( ) |
| 1. Feeling down, depressed, or hopeless | ( ) | ( ) | ( ) | ( ) | ( ) |
| 1. Feeling nervous, anxious or on edge | ( ) | ( ) | ( ) | ( ) | ( ) |
| 1. Not being able to stop or control worrying | ( ) | ( ) | ( ) | ( ) | ( ) |

Those with a combined score > 3 on items 1 & 2 (PHQ-2) complete remaining 6 items of the PHQ-8.

Those with a combined score > 3 on items 3 & 4 (GAD-2) complete remaining 5 items of the GAD-7.

**PHQ-8**

Over the past 2 weeks, how often have you been bothered by any of the following problems?

|  | **Not at all**  **(0)** | **Several days**  **(1)** | **More than half the days**  **(2)** | **Nearly every day**  **(3)** | **Decline to answer** |
| --- | --- | --- | --- | --- | --- |
| 1. Trouble falling or staying asleep, or sleeping too much? | ( ) | ( ) | ( ) | ( ) | ( ) |
| 1. Feeling tired or having little energy? | ( ) | ( ) | ( ) | ( ) | ( ) |
| 1. Poor appetite or overeating? | ( ) | ( ) | ( ) | ( ) | ( ) |
| 1. Feeling bad about yourself - or that you are a failure or have let yourself or your family down? | ( ) | ( ) | ( ) | ( ) | ( ) |
| 1. Trouble concentrating on things, such as reading the newspaper or watching television? | ( ) | ( ) | ( ) | ( ) | ( ) |
| 1. Moving or speaking so slowly that other people could have noticed, or the opposite - being so fidgety or restless that you have been moving around a lot more than usual? | ( ) | ( ) | ( ) | ( ) | ( ) |

**GAD-7**

Over the past 2 weeks, how often have you been bothered by any of the following problems?

|  | **Not at all**  **(0)** | **Several days**  **(1)** | **More than half the days**  **(2)** | **Nearly every day**  **(3)** | **Decline to answer** |
| --- | --- | --- | --- | --- | --- |
| 1. Worrying too much about different things? | ( ) | ( ) | ( ) | ( ) | ( ) |
| 1. Trouble relaxing? | ( ) | ( ) | ( ) | ( ) | ( ) |
| 1. Being so restless that it is hard to sit still? | ( ) | ( ) | ( ) | ( ) | ( ) |
| 1. Becoming easily annoyed or irritable? | ( ) | ( ) | ( ) | ( ) | ( ) |
| 1. Feeling afraid as if something awful might happen? | ( ) | ( ) | ( ) | ( ) | ( ) |

Resiliency –from Involvement

|  | Strongly disagree | Disagree | Neutral | Agree | Strongly Agree | Not Applicable |
| --- | --- | --- | --- | --- | --- | --- |
| I usually manage one way or another |  |  |  |  |  |  |
| I feel proud that I have accomplished things in my life |  |  |  |  |  |  |
| I usually take things in stride |  |  |  |  |  |  |
| I am friends with myself |  |  |  |  |  |  |
| I am determined |  |  |  |  |  |  |
| I keep interested in things |  |  |  |  |  |  |
| My belief in myself gets me through hard times |  |  |  |  |  |  |
| My life has meaning |  |  |  |  |  |  |
| When I am in a difficult situation, I can usually find my way out of it |  |  |  |  |  |  |
| I have enough energy to do what I have to do |  |  |  |  |  |  |

# Geospatial (using map)

*Intro: The next few questions will be using maps. We are collecting this information to assess average distances people are travelling. As with the rest of this survey, this information is kept confidential and your responses will not be associated with your name. Please drop a pin at the approximate location that is asked.*

- 1. Please drop a pin at the hospital or clinic that you might go to for primary care.
  2. Please drop a pin at your partner’s home. [Logic: only ask if reports a partner Q13]
  3. Please drop a pin at your work / school. [Logic: based on employment question Q10]

**Module 2**

# Basic HIV knowledge

(SHIPP & Sibanye)

| 29a. Do you know anyone who has HIV or AIDS? | ⬜ Yes | ⬜ No |
| --- | --- | --- |
| 30. A person who has HIV can look healthy | ⬜ True | ⬜ False |
| 31. If a person is infected with HIV, they show symptoms within a month of being infected. | ⬜ True | ⬜ False |
| 32. There is a vaccine that can stop you from getting HIV. | ⬜ True | ⬜ False |
| 33. Even if your partner has HIV, the risk for getting HIV is very low when deep kissing (tongue in partner’s mouth). | ⬜ True | ⬜ False |
| 34. Nearly all HIV transmission comes from having lots of boyfriends or hook-ups. | ⬜ True | ⬜ False |
| 35. The risk for getting HIV is very low when having oral sex | ⬜ True | ⬜ False |
| 36. A person is more likely to get HIV from receptive sex (bottom) than insertive sex (top). | ⬜ True | ⬜ False |
| 37. Showering or washing your genitals/private parts after having sex will make you less likely to get HIV. | ⬜ True | ⬜ False |

# PrEP Intentions

1. Were you considering going on PrEP before being a part of this study?
   1. Yes
   2. No
   3. Undecided
2. What are your reasons for choosing to take PrEP medication? Select all that apply. (UMMC Survey/AJS)
3. I don’t use condoms as often as I should
4. I don’t trust that using condoms alone will protect me
5. I want to be able to have sex without using condoms all the time
6. I’m worried that I will get HIV infection if I don’t take PrEP
7. My partner is HIV positive and wants me to take PrEP to protect myself
8. My doctor said I should take PrEP
9. If a PrEP home care service that had video visits with a doctor and self-administered HIV/STI testing from home, with new prescriptions mailed to you was widely available, would you?
   1. Prefer to use this service
   2. Prefer to visit a medical provider in-person every three months
   3. No preference

# PrEP Stigma

*Intro: Please indicate how much you agree with the following statements:*

Response options: 1= strongly agree, 2=agree, 3=neutral, 4= disagree, 5=strongly disagree

1. *Please indicate how much you agree with the following statements:Please indicate how much you agree with the following statements:*I would feel ashamed to take PrEP pills in front of others.
2. Someone taking PrEP should keep their pills hidden.
3. People experience negative judgment because they take PrEP.
4. I would have sex with someone who is taking PrEP.
5. Someone taking PrEP would be seen by others as slutty.
6. People taking PrEP receive praise for being responsible.
7. My *friends* would be supportive of me taking PrEP.
8. Someone taking PrEP would be treated unfairly by their doctors.
9. People experience problems when they tell their sex partner(s) they are taking PrEP.
10. I would feel proud to take PrEP every day.
11. People taking PrEP experience verbal harassment.
12. People on PrEP are taking care of their health.
13. My *family* would be supportive of me taking PrEP.

# Other sexual risk items specific to iTech:

1. In the **past 30 days**, has a partner harmed you physically, or attempted to harm you physically? This includes hitting you, kicking you, attempting to strangle you, and/or attacking you with a knife, gun or other weapon.

- Yes
- No
- Decline to answer

1. In the **past 30 days**, has a partner used physical force or threats of force to make you have sex when you did not want to?

- Yes
- No
- Decline to answer

1. In the **past 30 days**, has a partner harmed you emotionally, or attempted to harm you emotionally? This includes calling you names or putting you down.

- Yes
- No
- Decline to answer

1. If number of partners harmed >0 in recall period…..Of the partners who harmed you, did you have sex with any partners in exchange for something, like money, drugs, food, or a place to stay?

- Yes
- No
- Decline to answer

# Sexual Stigma

1. During the past 12 months, have any of the following things happened to you because someone knew or assumed you were attracted to men? (AMIS)

Response options: 1=No, 2=yes, 3=I prefer not to answer, 4=don’t know, 5= does not apply

- 1. You were called names or insulted
  2. You received poorer services than other people in restaurants, stores, other businesses or agencies
  3. You were treated unfairly at work or school
  4. You were denied or given lower quality health care
  5. You were physically attacked or injured

1. How strongly do you agree or disagree with the following statement: “Most people in my area are tolerant of gays and bisexuals”? (AMIS)

Response options: 1= strongly disagree, 2=disagree, 3=neutral, 4= agree, 5=strongly agree

# Perceived HIV risk (From SHIPP)

26. I think my chances of getting infected with HIV, in my lifetime, are: (sliding scale)

27. I think my chances of getting infected with HIV, in the next five years, are:

⬜ Almost zero

⬜ Small

⬜ Moderate

⬜ Large

⬜ Very large

28. Getting HIV infection is something I am:

⬜ Not concerned about

⬜ A little concerned about

⬜ Moderately concerned about

⬜ Concerned about a lot

⬜ Extremely concerned about

29. I am concerned about high rates of HIV infection among people like me.

⬜ Not concerned about

⬜ A little concerned about

⬜ Moderately concerned about

⬜ Concerned about a lot

⬜ Extremely concerned about

PrEP Use Self-Efficacy **(adapted from HIV-ASES**) (from From Al Liu)

We are going to ask you about situations that could occur while taking PrEP.

For the following questions we will ask you to tell us how confident you expect to do the following things.

Use this response scale ranging from 0 (“could not do at all”) to 10 (“completely certain could do”).

How confident are you that you could:

1) Take a PrEP pill every day even when side effects begin to interfere with daily activities

2) Integrate taking PrEP into your daily routine

4) Take a PrEP pill every day even when your daily routine is disrupted?

5) Take a PrEP pill every day when you aren’t feeling well?

6) Take a PrEP pill every day even when you don’t have a stable place to live?

7) Take a PrEP pill every day even when you are on a drug/alcohol binge?

8) Take a PrEP pill every day when if it could mean changing your eating habits?

10) Continue taking PrEP even when you are feeling discouraged about your health?

11) Continue taking PrEP every day even when getting to your clinic appointments is a major hassle?

12) Continue taking PrEP even when people close to you tell you that they don’t think that it is doing any good?

# Internalized Homophobia

1. Even if I could change my sexual orientation, I wouldn't.

- 1. Strongly Disagree
  2. Disagree
  3. Neither agree nor disagree
  4. Agree
  5. Strongly agree

2. Homosexuality is as natural as heterosexuality.

1. Strongly Disagree
2. Disagree
3. Neither agree nor disagree
4. Agree
5. Strongly agree
6. I feel comfortable being a man who has sex with men.
7. Strongly Disagree
8. Disagree
9. Neither agree nor disagree
10. Agree
11. Strongly agree
12. Social situations with gay men make me feel uncomfortable.
    1. Strongly Disagree
    2. Disagree
    3. Neither agree nor disagree
    4. Agree
    5. Strongly agree

5. I feel comfortable in gay bars.

1. Strongly Disagree
2. Disagree
3. Neither agree nor disagree
4. Agree
5. Strongly agree

6.I feel comfortable being seen in public with an obviously gay person.

- 1. Strongly Disagree
  2. Disagree
  3. Neither agree nor disagree
  4. Agree
  5. Strongly agree

7. I feel comfortable discussing homosexuality in a public setting.

1. Strongly Disagree
2. Disagree
3. Neither agree nor disagree
4. Agree
5. Strongly agree

# Health Literacy

1. How confident are you filling out medical forms for yourself? (SHIPP)

⬜ Not at all

⬜ A little bit

⬜ Somewhat

⬜ Quite a bit

⬜ Extremely

1. How often do you have someone help you read health materials?

⬜ Never (0)

⬜ Occasionally (1)

⬜ Sometimes (2)

⬜ Often (3)

⬜ Always (4)

1. How often do you find it hard to understand written information about your health? (adjusted SHIPP)

⬜ Never (0)

⬜ Occasionally (1)

⬜ Sometimes (2)

⬜ Often (3)

⬜ Always (4)

1. Do you need help to fill in official documents?
   1. Rarely
   2. Sometimes
   3. Often

# Outness to Primary Medical Care Provider

*A primary medical care provider is a health care provider that you see regularly, such as a pediatrician, a doctor, or other health care provider.*

1. Have you told your primary medical care provider that you have sex with men? (adjusted from ATN)
2. Yes
3. No
4. Decline to answer
5. If no, Have you told any medical provider that you have sex with men? Y/N/decline
6. When you are sick, do you seek out medical advice by visiting your doctor? Y/N/decline
7. Are you uncomfortable with seeking medical advice or assistance when it is needed because of your sexual orientation? Y/N/decline
8. Have you ever felt that you were treated poorly by a doctor or health care practitioner because of your sexual orientation? Y/N/decline
9. Have you found it difficult to find LGBT positive/affirming doctors medical practitioners? Y/N/decline

**Module 3**

# Substance Use/Abuse

1. In your life, have you ever used any of the following: *Select all that apply.*
   1. Tobacco (cigarettes, chewing tobacco, cigars, e-cigarettes, etc.)
   2. Alcohol (beer, wine, spirits, etc.)
   3. Marijuana (cannabis, pot, grass, hash, etc.) or synthetic cannabis
   4. Cocaine (coke, crack, etc.)
   5. Amphetamine-type stimulants (speed, meth, diet pills, ecstasy, Ritalin, Adderall, etc.)
   6. Inhalants (poppers, nitrous, glue, gas, paint thinner, etc.)
   7. Sedatives, tranquilizers, or sleeping pills (benzos, Valium, Ativan, Klonopin, Rohypnol, Xanax, Ambien, GHB, etc.)
   8. Hallucinogens (LSD, acid, mushrooms, PCP, Ketamine, etc.)
   9. Opioids (heroin, morphine, methadone, codeine, Oxycontin, Percocet, Vicodin, etc.)
   10. Steroids (anabolic steroids for body building)

**Only ask following questions that pertain to substance ppt clicked they used

| **Substance Use/Abuse** | | |
| --- | --- | --- |
| **Characteristic** | **Format/Values** | **Source/**  **Comments** |
| **TOBACCO_1: Lifetime use** In your life, have you ever used tobacco products (cigarettes, chewing tobacco, cigars, e-cigarettes, etc.)?  If **TOBACCO_1=No**  then skip to **ALCOHOL_1**. | Coded Fields: 1: Yes 2: No  Decline to answer | ATN 071 / Assist |
| **TOBACCO_2: Use in past 3 months** In the past 3 months, how often have you used tobacco products (cigarettes, chewing tobacco, cigars, e-cigarettes, etc.)?  If **TOBACCO_2 = Never**  then skip **TOBACCO_3-TOBACCO_5**. | Coded Fields:  1: Never 2: Once or Twice 3. Monthly 4: Weekly 5: Daily or Almost Daily  Decline to answer | ATN 071 / Assist |
| **TOBACCO_3: Desire to use in past 3 months** During the past 3 months, how often have you had a desire to use tobacco products (cigarettes, chewing tobacco, cigars, e-cigarettes, etc.)? | Coded Fields:  1: Never 2: Once or Twice 3. Monthly 4: Weekly 5: Daily or Almost Daily  Decline to answer | ATN 071 / Assist |
| **TOBACCO_4: Problems due to use in past 3 months** During the past 3 months, how often has your use of tobacco products (cigarettes, chewing tobacco, cigars, e-cigarettes, etc.) led to health, social, legal or financial problems? | Coded Fields:  1: Never 2: Once or Twice 3. Monthly 4: Weekly 5: Daily or Almost Daily  Decline to answer | ATN 071 / Assist |
| **TOBACCO_7: Ever attempted to reduce/stop use** Have you ever tried and failed to control, cut down or stop using tobacco products (cigarettes, chewing tobacco, cigars, e-cigarettes, etc.)? | Coded Fields: 1: No, Never 2: Yes, in the past 3 months 3: Yes, but not in the past 3 months  Decline to answer | ATN 071 / Assist |
| **ALCOHOL_1: Lifetime use** In your life, have you ever used alcoholic beverages (beer, wine, spirits, etc.)?  If **ALCOHOL_1=No**  then skip to **CANNABIS_1**. | Coded Fields: 1: Yes 2: No  Decline to answer | ATN 071 / Assist |
| **ALCOHOL_2.1: Drinks in a typical day**  How many drinks containing alcohol do you have on a typical day? | Coded Fields:  None  1: 1 or 2 2: 3 or 4 3. 5 or 6 4: 7 to 9 5: 10 or more  Decline to answer | AUDIT-C |
| **ALCOHOL_2.2: Binge Drinking**  How often do you have **[Insert Number]** or more drinks on one occasion?  **If participant sex assigned at birth is male, use 5 drinks. If female, use 4 drinks.** | Coded Fields:  1: Never  2: Less than monthly  3: Monthly  4: Weekly  5: Daily or almost daily  Decline to answer | Customized version of AUDIT-C question.  Reference: <http://www.niaaa.nih.gov/alcohol-health/overview-alcohol-consumption/moderate-binge-drinking> |
| **ALCOHOL_4: Problems due to use in past 3 months** During the past 3 months, how often has your use of alcoholic beverages (beer, wine, spirits, etc.) led to health, social, legal or financial problems? | Coded Fields:  1: Never 2: Once or Twice 3. Monthly 4: Weekly 5: Daily or Almost Daily  Decline to answer | ATN 071 / Assist |
| **ALCOHOL_7: Ever attempted to reduce/stop use** Have you ever tried and failed to control, cut down or stop using alcoholic beverages (beer, wine, spirits, etc.)? | Coded Fields: 1: No, Never 2: Yes, in the past 3 months 3: Yes, but not in the past 3 months  Decline to answer | ATN 071 / Assist |
| **Cannabis Introductory Script:**  In this section, we will ask you about cannabis and synthetic cannabis use. Please report on all use even if you have a prescription or cannabis use is legal in your state or area. | | |
| **CANNABIS_1: Lifetime use** In your life, have you ever used cannabis (marijuana, pot, grass, hash, etc.) or synthetic cannabis?  If **CANNABIS_1=No**  then skip to **COCAINE_1**. | Coded Fields: 1: Yes 2: No  Decline to answer | ATN 071 / Assist |
| **CANNABIS_1.1**  Do you have or have you had a prescription to use cannabis (marijuana, pot, grass, hash, etc.) or synthetic cannabis?  If **CANNABIS_1.1 = No**  then skip **CANNABIS_1.1.1.** | Coded Fields: 1: Yes 2: No  Decline to answer | Added per SUA working group. |
| **CANNABIS_2: Use in past 3 months** In the past 3 months, how often have you used cannabis (marijuana, pot, grass, hash, etc.) or synthetic cannabis?   If **CANNABIS_2 = Never**  then skip **CANNABIS_3-CANNABIS_5**. | Coded Fields:  1: Never 2: Once or Twice 3. Monthly 4: Weekly 5: Daily or Almost Daily  Decline to answer | ATN 071 / Assist |
| **CANNABIS_4: Problems due to use in past 3 months** During the past 3 months, how often has your use of cannabis (marijuana, pot, grass, hash, etc.) or synthetic cannabis led to health, social, legal or financial problems? | Coded Fields:  1: Never 2: Once or Twice 3. Monthly 4: Weekly 5: Daily or Almost Daily  Decline to answer | ATN 071 / Assist |
| **CANNABIS_7: Ever attempted to reduce/stop use** Have you ever tried and failed to control, cut down or stop using cannabis (marijuana, pot, grass, hash, etc.) or synthetic cannabis? | Coded Fields: 1: No, Never 2: Yes, in the past 3 months 3: Yes, but not in the past 3 months  Decline to answer | ATN 071 / Assist |
| **COCAINE_1: Lifetime use** In your life, have you ever used cocaine (coke, crack, etc.)?  If **COCAINE_1=No**  then skip to **AMPHETAMINE_1**. | Coded Fields: 1: Yes 2: No  Decline to answer | ATN 071 / Assist |
| **COCAINE_2: Use in past 3 months** In the past 3 months, how often have you used cocaine (coke, crack, etc.)?  If **COCAINE_2 = Never**  then skip **COCAINE_3-COCAINE_5**. | Coded Fields:  1: Never 2: Once or Twice 3. Monthly 4: Weekly 5: Daily or Almost Daily  Decline to answer | ATN 071 / Assist |
| **COCAINE_3: Desire to use in past 3 months** During the past 3 months, how often have you had a desire to use cocaine (coke, crack, etc.)? | Coded Fields:  1: Never 2: Once or Twice 3. Monthly 4: Weekly 5: Daily or Almost Daily  Decline to answer | ATN 071 / Assist |
| **COCAINE_4: Problems due to use in past 3 months** During the past 3 months, how often has your use of cocaine (coke, crack, etc.) led to health, social, legal or financial problems? | Coded Fields:  1: Never 2: Once or Twice 3. Monthly 4: Weekly 5: Daily or Almost Daily  Decline to answer | ATN 071 / Assist |
| **COCAINE_7: Ever attempted to reduce/stop use** Have you ever tried and failed to control, cut down or stop using cocaine (coke, crack, etc.)? | Coded Fields: 1: No, Never 2: Yes, in the past 3 months 3: Yes, but not in the past 3 months  Decline to answer | ATN 071 / Assist |
| **Amphetamine Introductory Script:**  In this section, we will ask you about amphetamine use. Please report on all non-prescription drug use. Please report on use of prescription amphetamines only if you have taken such medications for reasons other than prescription, or taken them more frequently or at higher doses than prescribed. | | |
| **AMPHETAMINE_1: Lifetime use** In your life, have you ever used amphetamine type stimulants (speed, meth, diet pills, ecstasy, Ritalin, Adderall, etc.)?  If **AMPHETAMINE_1=No**  then skip to **INHALANTS_1**. | Coded Fields: 1: Yes 2: No  Decline to answer | ATN 071 / Assist |
| **AMPHETAMINE_2: Use in past 3 months** In the past 3 months, how often have you used amphetamine type stimulants (speed, meth, diet pills, ecstasy, Ritalin, Adderall, etc.)?  If **AMPHETAMINE_2 = Never**  then skip **AMPHETAMINE_3-AMPHETAMINE_5**. | Coded Fields:  1: Never 2: Once or Twice 3. Monthly 4: Weekly 5: Daily or Almost Daily  Decline to answer | ATN 071 / Assist |
| **AMPHETAMINE_3: Desire to use in past 3 months** During the past 3 months, how often have you had a desire to use amphetamine type stimulants (speed, meth, diet pills, ecstasy, Ritalin, Adderall, etc.)? | Coded Fields:  1: Never 2: Once or Twice 3. Monthly 4: Weekly 5: Daily or Almost Daily  Decline to answer | ATN 071 / Assist |
| **AMPHETAMINE_4: Problems due to use in past 3 months** During the past 3 months, how often has your use of amphetamine type stimulants (speed, meth, diet pills, ecstasy, Ritalin, Adderall, etc.) led to health, social, legal or financial problems? | Coded Fields:  1: Never 2: Once or Twice 3. Monthly 4: Weekly 5: Daily or Almost Daily  Decline to answer | ATN 071 / Assist |
| **AMPHETAMINE_7: Ever attempted to reduce/stop use** Have you ever tried and failed to control, cut down or stop using amphetamine type stimulants (speed, meth, diet pills, ecstasy, Ritalin, Adderall, etc.)? | Coded Fields: 1: No, Never 2: Yes, in the past 3 months 3: Yes, but not in the past 3 months  Decline to answer | ATN 071 / Assist |
| **INHALANTS_1: Lifetime use** In your life, have you ever used inhalants (poppers, nitrous, glue, gas, paint thinner, etc.)?  If **INHALANTS_1=No**  then skip to **SEDATIVES_1**. | Coded Fields: 1: Yes 2: No  Decline to answer | ATN 071 / Assist |
| **INHALANTS_2: Use in past 3 months** In the past 3 months, how often have you used inhalants (poppers, nitrous, glue, gas, paint thinner, etc.)?  If **INHALANTS_2 = Never**  then skip **INHALANTS_3-INHALANTS_5**. | Coded Fields:  1: Never 2: Once or Twice 3. Monthly 4: Weekly 5: Daily or Almost Daily  Decline to answer | ATN 071 / Assist |
| **INHALANTS_3: Desire to use in past 3 months** During the past 3 months, how often have you had a desire to use inhalants (poppers, nitrous, glue, gas, paint thinner, etc.)? | Coded Fields:  1: Never 2: Once or Twice 3. Monthly 4: Weekly 5: Daily or Almost Daily  Decline to answer | ATN 071 / Assist |
| **INHALANTS_4: Problems due to use in past 3 months** During the past 3 months, how often has your use of inhalants (poppers, nitrous, glue, gas, paint thinner, etc.) led to health, social, legal or financial problems? | Coded Fields:  1: Never 2: Once or Twice 3. Monthly 4: Weekly 5: Daily or Almost Daily  Decline to answer | ATN 071 / Assist |
| **INHALANTS_7: Ever attempted to reduce/stop use** Have you ever tried and failed to control, cut down or stop using inhalants (poppers, nitrous, glue, gas, paint thinner, etc.)? | Coded Fields: 1: No, Never 2: Yes, in the past 3 months 3: Yes, but not in the past 3 months  Decline to answer | ATN 071 / Assist |
| **Sedative Introductory Script:**  In this section, we will ask you about sedative, tranquilizer, and sleeping pills use. Please report on all non-prescription drug use. Please report on use of prescription drugs only if you have taken such medications for reasons other than prescription, or taken them more frequently or at higher doses than prescribed. | | |
| **SEDATIVES_1: Lifetime use** In your life, have you ever used sedatives, tranquilizers, or sleeping pills (benzos, Valium, Rohypnol, Xanax, Ambien, GHB, etc.)?  If **SEDATIVES_1=No**  then skip to **HALLUCINOGENS_1**. | Coded Fields: 1: Yes 2: No  Decline to answer | ATN 071 / Assist |
| **SEDATIVES_2: Use in past 3 months** In the past 3 months, how often have you used sedatives, tranquilizers, or sleeping pills (benzos, Valium, Rohypnol, Xanax, Ambien, GHB, etc.)?  If **SEDATIVES_2 = Never**  then skip **SEDATIVES_3-SEDATIVES_5**. | Coded Fields:  1: Never 2: Once or Twice 3. Monthly 4: Weekly 5: Daily or Almost Daily  Decline to answer | ATN 071 / Assist |
| **SEDATIVES_3: Desire to use in past 3 months** During the past 3 months, how often have you had a desire to use sedatives, tranquilizers, or sleeping pills (benzos, Valium, Rohypnol, Xanax, Ambien, GHB, etc.)? | Coded Fields:  1: Never 2: Once or Twice 3. Monthly 4: Weekly 5: Daily or Almost Daily  Decline to answer | ATN 071 / Assist |
| **SEDATIVES_4: Problems due to use in past 3 months** During the past 3 months, how often has your use of sedatives, tranquilizers, or sleeping pills (benzos, Valium, Rohypnol, Xanax, Ambien, GHB, etc.) led to health, social, legal or financial problems? | Coded Fields:  1: Never 2: Once or Twice 3. Monthly 4: Weekly 5: Daily or Almost Daily  Decline to answer | ATN 071 / Assist |
| **SEDATIVES_7: Ever attempted to reduce/stop use** Have you ever tried and failed to control, cut down or stop using sedatives, tranquilizers, or sleeping pills (benzos, Valium, Rohypnol, Xanax, Ambien, GHB, etc.)? | Coded Fields: 1: No, Never 2: Yes, in the past 3 months 3: Yes, but not in the past 3 months  Decline to answer | ATN 071 / Assist |
| **HALLUCINOGENS_1: Lifetime use** In your life, have you ever used hallucinogens (LSD, acid, mushrooms, PCP, Ketamine, etc.)?  If **HALLUCINOGENS_1=No**  then skip to **OPIOIDS_1**. | Coded Fields: 1: Yes 2: No  Decline to answer | ATN 071 / Assist |
| **HALLUCINOGENS_2: Use in past 3 months** In the past 3 months, how often have you used hallucinogens (LSD, acid, mushrooms, PCP, Ketamine, etc.)?  If **HALLUCINOGENS_2 = Never**  then skip **HALLUCINOGENS_3-HALLUCINOGENS_5**. | Coded Fields:  1: Never 2: Once or Twice 3. Monthly 4: Weekly 5: Daily or Almost Daily  Decline to answer | ATN 071 / Assist |
| **HALLUCINOGENS_3: Desire to use in past 3 months** During the past 3 months, how often have you had a desire to use hallucinogens (LSD, acid, mushrooms, PCP, Ketamine, etc.)? | Coded Fields:  1: Never 2: Once or Twice 3. Monthly 4: Weekly 5: Daily or Almost Daily  Decline to answer | ATN 071 / Assist |
| **HALLUCINOGENS_4: Problems due to use in past 3 months** During the past 3 months, how often has your use of hallucinogens (LSD, acid, mushrooms, PCP, trips, Ketamine, etc.) led to health, social, legal or financial problems? | Coded Fields:  1: Never 2: Once or Twice 3. Monthly 4: Weekly 5: Daily or Almost Daily  Decline to answer | ATN 071 / Assist |
| **HALLUCINOGENS_7: Ever attempted to reduce/stop use** Have you ever tried and failed to control, cut down or stop using hallucinogens (LSD, acid, mushrooms, PCP, Ketamine, etc.)? | Coded Fields: 1: No, Never 2: Yes, in the past 3 months 3: Yes, but not in the past 3 months  Decline to answer | ATN 071 / Assist |
| **Opioid Introductory Script:**  In this section, we will ask you about opioid use. Please report on use of all non-prescription drugs. Please report on use of prescription drugs only if you have taken such medications for reasons other than prescription, or taken them more frequently or at higher doses than prescribed. | | |
| **OPIOIDS_1: Lifetime use** In your life, have you ever used opioids (heroin, morphine, methadone, codeine, Oxycontin, Percocet, Vicodin, etc.)?  If **OPIOIDS_1=No**  then skip to **END**. | Coded Fields: 1: Yes 2: No  Decline to answer | ATN 071 / Assist |
| **OPIOIDS_2: Use in past 3 months** In the past 3 months, how often have you used opioids (heroin, morphine, methadone, codeine, Oxycontin, Percocet, Vicodin, etc.)?  If **OPIOIDS_2 = Never**  then skip **OPIOIDS_2.1-OPIOIDS_5**. | Coded Fields: 1: Never 2: Once or Twice 3. Monthly 4: Weekly 5: Daily or Almost Daily  Decline to answer | ATN 071 / Assist |
| **OPIOIDS_2.1: Heroin use in the past 3 months**  In the past 3 months, has your opioid use included heroin?  If **OPIOIDS_2.1 = No**  then skip **OPIOIDS_2.2**. | Coded Fields: 1: Yes 2: No  Decline to answer |  |
| **OPIOIDS_2.2: Heroin as primary opioid**  In the past 3 months, was heroin the most common opioid that you used? | Coded Fields: 1: Yes 2: No  Decline to answer |  |
| **OPIOIDS_3: Desire to use in past 3 months** During the past 3 months, how often have you had a desire to use opioids (heroin, morphine, methadone, codeine, Oxycontin, Percocet, Vicodin, etc.)? | Coded Fields:  1: Never 2: Once or Twice 3. Monthly 4: Weekly 5: Daily or Almost Daily  Decline to answer | ATN 071 / Assist |
| **OPIOIDS_4: Problems due to use in past 3 months** During the past 3 months, how often has your use of opioids (heroin, morphine, methadone, codeine, Oxycontin, Percocet, Vicodin, etc.) led to health, social, legal or financial problems? | Coded Fields:  1: Never 2: Once or Twice 3. Monthly 4: Weekly 5: Daily or Almost Daily  Decline to answer | ATN 071 / Assist |
| **OPIOIDS_7: Ever attempted to reduce/stop use** Have you ever tried and failed to control, cut down or stop using opioids (heroin, morphine, methadone, codeine, Oxycontin, Percocet, Vicodin, etc.)? | Coded Fields: 1: No, Never 2: Yes, in the past 3 months 3: Yes, but not in the past 3 months  Decline to answer | ATN 071 / Assist |
| **Steroid Introductory Script:**  In this section, we will ask you about steroid use. Please report on use of all non-prescription drugs. Please report on use of prescription drugs only if you have taken such medications for reasons other than prescription, or taken them more frequently or at higher doses than prescribed. | | |
| **STEROIDS_1: Lifetime use** In your life, have you ever used steroids (anabolic steroids for body building)?  If **STEROIDS_1=No**  then skip to **END**. | Coded Fields: 1: Yes 2: No  Decline to answer | ATN 071 / Assist |
| **STEROIDS_2: Use in past 3 months** In the past 3 months, how often have you used steroids (anabolic steroids for body building)?  If **STEROIDS_2 = Never**  then skip **STEROIDS_2.1-STEROIDS_5**. | Coded Fields: 1: Never 2: Once or Twice 3. Monthly 4: Weekly 5: Daily or Almost Daily  Decline to answer | ATN 071 / Assist |
| **STEROIDS_4: Problems due to use in past 3 months** During the past 3 months, how often has your use of steroids (anabolic steroids for body building)? | Coded Fields:  1: Never 2: Once or Twice 3. Monthly 4: Weekly 5: Daily or Almost Daily  Decline to answer | ATN 071 / Assist |
| **STEROIDS_7: Ever attempted to reduce/stop use** Have you ever tried and failed to control, cut down or stop using steroids (anabolic steroids for body building)? | Coded Fields: 1: No, Never 2: Yes, in the past 3 months 3: Yes, but not in the past 3 months  Decline to answer | ATN 071 / Assist |

# Injection Behaviors

1. Have you ever injected any drugs other than those prescribed for you? We mean anytime you might have used drugs with a needle, either by mainlining, skin popping, or muscling.
   1. Yes
   2. No
   3. Don’t know
2. [If Yes or Don’t know] In the **past 12 months**, on average, how often do you inject?
   1. More than once a day
   2. Once a day
   3. More than once a week
   4. Once a week
   5. More than once a month
   6. Once a month
   7. Less than once a month
   8. Never
   9. Don’t know
3. In the **past 6 months**, have you injected by using needles, syringes, or other drug preparation equipment (works) that had already been used by another person? (CDC)
   1. Yes
   2. No
4. Have you been screened for Hep C? [Logic: if ever injected]
   1. Yes, tested negative for Hep C
   2. Yes, tested positive for Hep C
   3. No, never tested
   4. Don’t know

# CAGE-AID Questions

1. Have you ever felt that you ought to cut down on your drinking or drug use?
   1. Yes
   2. No
2. Have people annoyed you by criticizing your drinking or drug use?
   1. Yes
   2. No
3. Have you ever felt bad or guilty about your drinking or drug use?
   1. Yes
   2. No
4. Have you ever had a drink or used drugs first thing in the morning to steady your nerves or to get rid of a hangover?
   1. Yes
   2. No

# Religiosity

1. How often do you attend religious or spiritual services?
   1. Never
   2. Holidays
   3. Monthly
   4. Weekly
   5. Daily
2. How accepted do you feel (as a man who has sex with men) by your religious or spiritual community?
   1. No acceptance
   2. Some acceptance
   3. Moderate acceptance
   4. High acceptance
   5. Complete acceptance
   6. Do not know
3. How often do you consult a spiritual or religious leader?
   1. Never
   2. Occasionally
   3. Monthly
   4. Weekly
   5. Daily

# Technology Use Questions

(General iTech specific--Mandatory)

1. Which of the following devices do you own? (Check all that apply)
   - Cell phone (basic mobile phone for calling or texting; does not have internet access, apps, or a touch screen)
   - Smartphone (advanced mobile phone with internet access, apps, and a touch screen)
   - Desktop computer
   - Laptop computer
   - Tablet computer
   - E-book reader
   - Fitness tracker or smart watch
   - Another device, specify: _______________________
   - Decline to answer
2. How do you usually access the internet? (Check all that apply)
   - Smartphone
   - Desktop computer
   - Laptop computer
   - Tablet computer
   - E-book reader
   - Another method, specify: _______________________
   - Decline to answer

If “Smartphone” is selected in #5:

1. What operating system/platform does your primary smartphone use?

- Apple (iOS)
- Android
- Windows
- Samsung
- BlackBerry
- Series 40 (S40)
- SymbianOS
- Don’t know
- Other, specify:
- Decline to answer

If “Cell phone” and/or “Smartphone” is selected in #5:

1. Do you regularly share your [phone response(s) from #5] with one or more other people (such as a partner, family member, or friends)?

- Yes
- No
- Decline to answer

If “Cell phone” and/or “Smartphone” is selected in #1:

1. What kind of [phone response(s) from #5] service do you have?

I have a prepaid account

I have a monthly [phone response(s) from #5] contract

I’m on a shared plan

Don’t know

None of the above

Decline to answer

If “Cell phone” and/or “Smartphone” is selected in #5:

1. Who pays for your [phone response(s) from #5] plan?

I do

Someone else does

Don’t know

Decline to answer

1. In the past year, was your phone service ever disconnected because you could not pay the bill or because your phone was lost or stolen?

- Yes
- No
- Decline to answer

If #11 = Yes:

1. How many times in the last 12 months has your phone been disconnected?

- Once
- Twice
- 3 to 5 times
- More than 5 times
- Decline to answer

If #11 = Yes:

1. The *last* time your phone was disconnected, for how long was it disconnected?

- 1 day or less
- 2 to 7 days
- 1 to 4 weeks
- 1 month or more
- Decline to answer

1. On average, how many hours *per day* do you spend on the internet (online) outside of your school or work responsibilities?

- No hours
- Less than an hour
- 1 to 3 hours
- 4 to 6 hours
- 7 to 9 hours
- 10 to 12 hours
- 13 to 15 hours
- 16 hours or more
- Decline to answer

If “Smartphone” is selected in #5:

1. How often do you use apps on your smartphone (for example: Facebook, dating apps, banking apps, Snapchat)?
   - More than once a day
   - About once a day
   - A few times a week
   - About once a week
   - Less than once a week
   - I do not use apps on my phone
   - Decline to answer
2. How often do you use websites [(if “Smartphone” is selected in #5) or smartphone apps] for the following reasons:

|  | Never | Rarely | Sometimes | Often | Decline to answer |
| --- | --- | --- | --- | --- | --- |
| Find a date |  |  |  |  |  |
| Meet partners for sex |  |  |  |  |  |
| Track your health behaviors (diet, exercise, medication management, etc.) |  |  |  |  |  |
| Get information about HIV or other STDs |  |  |  |  |  |
| Get other health or medical information |  |  |  |  |  |

*OPTIONAL ATN QUESTIONS:*

1. Do you have an active account on any of the following social networking sites?

- Facebook
- Instagram
- Twitter
- Tumblr
- Snapchat
- Grindr
- Scruff
- Jack’d
- Hornet
- Chappy
- Tinder
- Surge
- GROWLr
- OkCupid
- Adam4Adam
- BGC Live
- Manhunt
- Plenty of Fish
- Squirt
- Other, please specify: _____________________
- Decline to answer

If “Find a date” != never in #16:

1. During the past 30 days, how often did you use websites [if “Smartphone” is selected in #5) or smartphone apps] to find someone to date (a potential romantic relationship)?

- Never
- Once a month or less
- 2-3 times a month
- About once a week
- 2-6 times a week
- About once a day
- Decline to answer

If “Meet partners for casual sex” != never in #16:

1. During the past 30 days, how many times did you use websites [if “Smartphone” is selected in #5) or smartphone apps] to meet partners for casual sex (have no-strings attached sex)?

- Never
- Once a month or less
- 2-3 times a month
- About once a week
- 2-6 times a week
- About once a day
- Decline to answer

If “Find a date” AND “Meet partners for casual sex” != never in #16

***Thank you for completing the behavioral assessment for ePrEP!***
